# Detecting and Remediating Harmful Data Shifts for the Responsible Deployment of Clinical AI Models

**DOI:** 10.1001/jamanetworkopen.2025.13685

**Published:** 2025-06-04

**Authors:** Vallijah Subasri, Amrit Krishnan, Ali Kore, Azra Dhalla, Deval Pandya, Bo Wang, David Malkin, Fahad Razak, Amol A. Verma, Anna Goldenberg, Elham Dolatabadi

**Affiliations:** 1 Peter Munk Cardiac Centre, University Health Network, Toronto, Ontario, Canada; 2 Vector Institute for Artificial Intelligence, Toronto, Ontario, Canada; 3 Genetics and Genome Biology Program, The Hospital for Sick Children, Toronto, Ontario, Canada; 4 Department of Computer Science, University of Toronto, Toronto, Ontario, Canada; 5 Division of Hematology/Oncology, The Hospital for Sick Children, Toronto, Ontario, Canada; 6 Department of Pediatrics, University of Toronto, Toronto, Ontario, Canada; 7 St Michael’s Hospital, Unity Health Toronto, Toronto, Ontario, Canada; 8 Institute of Health Policy, Management and Evaluation, University of Toronto, Toronto, Ontario, Canada; 9 Department of Medicine, University of Toronto, Toronto, Ontario, Canada; 10 Department of Laboratory Medicine and Pathobiology, University of Toronto, Toronto, Ontario, Canada; 11 Faculty of Health, York University, Toronto, Ontario, Canada

## Abstract

**Importance:**

Clinical artificial intelligence (AI) systems are susceptible to performance degradation due to data shifts, which can lead to erroneous predictions and potential patient harm. Proactively detecting and mitigating these shifts is crucial for maintaining AI effectiveness and safety in clinical practice.

**Objectives:**

To develop and evaluate a proactive, label-agnostic monitoring pipeline to detect and mitigate harmful data shifts in clinical AI systems and to assess the use of transfer learning and continual learning strategies in maintaining model performance.

**Design, Setting, and Participants:**

This prognostic study was conducted using electronic health record data for admissions to general internal medicine wards of 7 large hospitals (5 academic and 2 community) in Toronto, Canada, between January 1, 2010, to August 31, 2020. Inpatients (aged ≥18 years) with a hospital stay of at least 24 hours were included. Data analysis was performed from January to August 2022.

**Exposures:**

Data shifts due to changes in hospital type, critical laboratory assays, patient demographics, admission type, and the COVID-19 pandemic.

**Main Outcomes and Measures:**

The primary outcome was predictive performance for all-cause in-hospital mortality within the next 2 weeks, evaluated using the area under the receiver operating characteristic curve (AUROC) and the area under the precision-recall curve (AUPRC). Data shifts were detected using a label-agnostic monitoring pipeline employing a black box shift estimator with maximum mean discrepancy testing.

**Results:**

Data were available for 143 049 adult inpatients (mean [SD] age, 67.8 [19.6] years; 50.7% female). Significant data shifts were detected as a result of changes in younger age groups and admissions from nursing homes and acute care centers, transferring from community to academic hospitals, and changes in brain natriuretic peptide and D-dimer. Transfer learning improved model performance of community hospitals in a hospital type–dependent manner (Delta AUROC [SD], 0.05 [0.03]; Delta AUPRC [SD], 0.06 [0.04]). During the COVID-19 pandemic, drift-triggered continual learning improved overall model performance (Delta AUROC [SD], 0.44 [0.02]; *P* = .007, Mann-Whitney *U* test).

**Conclusions and Relevance:**

In this prognostic study, a proactive, label-agnostic monitoring pipeline detected harmful data shifts for a clinical AI system predicting in-hospital mortality. Transfer learning and drift-triggered continual learning strategies mitigated performance degradation, maintaining model performance across health care settings. These findings suggest that the approach used here may ensure the robust and equitable deployment of clinical AI models. Future research should explore the generalizability of this framework across diverse clinical domains, data modalities, and longer deployment periods to further validate its effectiveness.

## Key Points

**Question:**

Can harmful data shifts in clinical artificial intelligence (AI) models be proactively detected in a label-agnostic manner, and can transfer and continual learning strategies help mitigate model deterioration across health care settings?

**Findings:**

In this prognostic study of 143 049 patients across 7 hospitals in Toronto, Canada, significant data shifts due to changes in demographics, hospital types, admission sources, and critical laboratory assays were detected using a label-agnostic monitoring pipeline. Transfer learning and drift-triggered continual learning strategies mitigated these shifts, significantly improving model performance during the COVID-19 pandemic.

**Meaning:**

These findings suggest that a proactive, label-agnostic monitoring pipeline incorporating transfer and continual learning can detect and mitigate harmful data shifts in Toronto’s general internal medicine population, ensuring robust and equitable clinical AI deployment.

## Teaser

This prognostic study uses hospital admission data to evaluate a proactive, label-agnostic monitoring pipeline incorporating transfer learning and continual learning strategies to detect and mitigate harmful data shifts in clinical artificial intelligence (AI) systems.

## Introduction

Artificial intelligence (AI) systems have shown promise in predicting clinical outcomes including mortality,^1,2,3^ length of stay,^4^ sepsis,^5,6,7^ and specific disease diagnoses.^8^ However, a notable misalignment exists between the potential promised during the development of AI models and their deployment in clinical health care settings,^9^ with benefits of improving clinical outcomes and reducing costs not yet realized.^10,11^ A key barrier hindering the realization of this potential is system malfunction caused by data shifts,^12,13^ occurring when training data (source) differ significantly from evaluation data (target). The term *data shift* refers to changes in the joint distribution of data between model training and deployment,^14^ whereas *data drift* refers specifically to gradual time-dependent changes in data distributions.^15^ In health care, these shifts can exist along the axes of institutional differences (eg, staffing, instruments, and data-collection workflows), epidemiologic changes (eg, diseases or catastrophic events),^16^ behavioral shifts (eg, reimbursement incentives or changes in clinical practice),^17^ or differences in patient demographics (eg, race and ethnicity, sex, age, or socioeconomic status).^13,18,19,20^

Harmful data shifts can lead to the perpetuation of algorithmic biases, missing critical diagnoses, and unnecessary clinical interventions that can be detrimental to patient outcomes and burden the health care system.^10,11,21^ AI systems are constantly subject to data bias from variability in the social patterning of data generation, including changes in clinical care and measurement practices of critical devices,^22^ such as catheters,^23^ pulse oximeters,^24,25^ temperature monitors,^26^ and blood pressure monitors.^27^ Studies show limited generalizability across demographics,^18,28^ countries,^29^ hospitals,^30^ and time periods.^16,31^ The challenge of obtaining timely ground-truth labels for clinical outcomes, such as mortality, suggests that model updating based on recognizing deterioration in model performance is impractical and necessitates label-agnostic shift detection.^32,33,34^

We developed a pipeline to identify harmful data shifts, ensuring the readiness for deployment^35^ of a clinical AI model for in-hospital mortality prediction. We used transfer learning^30^ to improve cross-site model performance^36,37,38^ and continual learning to proactively update our model in the presence of data shifts.

## Methods

### Study Cohort

We conducted this prognostic study using deidentified electronic health record (EHR) data from patients admitted to general internal medicine (GIM) wards from January 1, 2010, to August 31, 2020, across 7 hospitals in Toronto, Canada. Inpatients (aged ≥18 years) with a hospital stay of at least 24 hours were included. Participating hospitals included Mount Sinai Hospital, Toronto General Hospital, Toronto Western Hospital, Trillium Health Partners (THP) Mississauga, THP Credit Valley, St Michael’s Hospital, and Sunnybrook Hospital. In keeping with the conventions of the General Medicine Inpatient Initiative (GEMINI) to avoid the disclosure of sensitive hospital information, we have anonymized hospital names. All patient data were collected and approved through GEMINI under the oversight of the research ethics board (REB) at Toronto Academic Health Science Network.^39,40^ The REB extension was approved by Unity Health Toronto. A separate REB approval was obtained for THP. Informed consent was waived because the study relied solely on deidentified patient data and obtaining consent from more than 1 million discharged patients is impracticable (further details on GEMINI’s data protection practices are available online^41^). The study followed the Transparent Reporting of a Multivariable Prediction Model for Individual Prognosis or Diagnosis (TRIPOD+AI) reporting guideline.

### Outcome Measures

The primary outcome was the predictive performance for all-cause in-hospital mortality within the next 2 weeks, to ensure sufficient positive labels (eFigure 1 in Supplement 1). The outcome reflects any in-hospital mortality event, including intensive care unit deaths, but does not capture postdischarge mortality. Labels were encoded as 1 if a patient died within 2 weeks and 0 if the patient was alive. The prediction was made every 24 hours, starting 24 hours after admission, using the target replication approach (eFigure 2 in Supplement 1).^42^

### Data Preprocessing

The base model consisted of 91 features comprising laboratory tests, blood transfusions, imaging reports, and administrative features (eTable 1 in Supplement 1); a rigorous internal validation process^40^ demonstrated 98% to 100% accuracy across key data types. The base plus comorbidities model consisted of the 91 features used in our base model in addition to 18 comorbidities based on the Charlson Comorbidity Index.^43^ The base plus diagnosis codes model consisted of the 91 features used in our base model in addition to the 22 groupings of *International Statistical Classification of Diseases, Tenth Revision* (*ICD-10*) diagnosis codes. The input features used for time-series modeling were aggregated by taking the mean for 24-hour timesteps over 144 hours. Missing values were imputed using forward filling, followed by backward filling.

### Model Development

We simulated deployment using an 8:2 random split of data from 2010 to 2018 for training and validation and data from 2019 to 2020 for testing. Reported metrics reflect performance on the test set, which was strictly time-separated to prevent data leakage and preserve clinical applicability. Time-series models including a recurrent neural network, gated recurrent unit (GRU), and long short-term memory (LSTM) were leveraged because they are better at capturing long-term dependencies,^42^ with LSTM performing best (eTable 2 in Supplement 1). Models were trained using PyTorch, version 1.13.1 (Linux Foundation) and optimized for binary cross-entropy using the Adagrad adaptive gradient algorithm^44^ with a batch size of 64, a step size of 128, and γ of 0.5, and hyperparameters were tuned (eTable 3 in Supplement 1). To account for class imbalance, we reweighted the loss by the fraction of control patients/case patients. Each model was trained with early stopping using patience of 3, a Delta of 0, and the sigmoid activation function for prediction probabilities. SEs were calculated over 5 repetitions with random weight initializations. The optimal configuration consisted of 2 hidden layers, 128 hidden cells, dropout of 0.2, learning rate of 1.0 × 10^−4^, and weight decay of 1.0 × 10^−7^. Model parameters were fixed throughout experiments.

### Monitoring and Evaluation Pipeline

We developed a monitoring and evaluation pipeline consisting of (1) shift application, (2) dimensionality reduction, (3) statistical testing, (4) sensitivity testing, and (5) rolling window analysis. First, EHR data were split into source and target datasets based on clinical data shift experiments (eg, hospital type, time periods). Second, softmax outputs of an LSTM label classifier were trained on source data (black box shift estimator [BBSE]).^45^ Third, 2-sample testing (1-sided) was conducted using maximum mean discrepancy (MMD) to detect data shifts between source and target data. Fourth, data shift detection was conducted across increasing target data sample sizes. Finally, a 14-day rolling window was used to assess data drift because the average length of stay in our cohort was just under 2 weeks (eFigure 1 in Supplement 1) and because it optimally balances case volume, responsiveness, and alignment with clinical work cycles.^46^

To determine the optimal combination of dimensionality reduction and statistical testing, we benchmarked on synthetic shifts: gaussian noise (eTables 4 and 5 in Supplement 1) and feature swap (eTable 6 in Supplement 1). We evaluated varying dimensionality reduction (ie, sparse random projections, principal component analysis [PCA], kernel PCA, or BBSE), statistical tests (ie, Kolmogorov-Smirnov test, MMD, or χ^2^), and model-based tests (ie, classifier, spot-the-difference). Based on the synthetic experiments, we found that BBSE plus MMD was the optimal choice for the shift detector. To evaluate performance, we used the area under the receiver operating characteristic curve (AUROC), the area under the precision-recall curve (AUPRC), sensitivity, and positive predictive value.

### Clinical Data Shift Experiments

We devised data splits reflecting clinical scenarios that may result in harmful data shifts, using an 8:2 train-test. An LSTM model was trained on 80% of source data, with a shift detector (fit on 10 000 random patients) evaluating on 20% of in-distribution source test data and out-of-distribution target data. We examined general patient characteristics (age [18-29, 30-44, 45-64, or ≥65 years], sex [male or female], and admission source [acute care or nursing home], with possible source-target overlap), location-based shifts (academic hospital or community hospital), and time-based shifts (COVID-19 pandemic and laboratory assay changes in troponin, brain natriuretic peptide [BNP], and D-dimer). Experiments are outlined in the eMethods in Supplement 1, with sample sizes detailed in eTable 7 in Supplement 1.

### Training Strategies for Cross-Site Deployment

We evaluated training strategies using 6:2:2 train-validation-test splits for hospital type–specific models (academic or community) and using the all-hospital model. Strategies included pretraining only, fine-tuning (freezing all but the final layer for 1 or 10 epochs), and single-hospital ablation, with performance evaluated on test data from each hospital.

### Prospective Evaluation and Model Updating

We simulated prospective evaluation using daily assessments (stride = 1) with a 14-day rolling window from March 1, 2019, to August 31, 2020. Continual learning strategies included periodic updating (at 7- to 60-day intervals), data shift–triggered most-recent updating (using 7-270 days of recent data), and data shift–triggered cumulative updating (using data to date). Model updating was optimized for updating window size, lookback window, *P* value threshold, sample size for shift detection, and number of epochs. We also compared sampling strategies where we used (1) all of the encounters, (2) correctly predicted encounters, and (3) positively predicted encounters.

### Statistical Analysis

Statistical analysis was conducted with Python, version 3.10.4 (Python Software Foundation) using SciPy, version 1.11.3. *P* < .05 (left-tailed) was considered statistically significant for performance metrics, and *P* < .05 (right-tailed) was considered statistically significant for data shift metrics. Data analysis was performed between January and August 2022.

## Results

### Subgroup Analyses

**Figure 1.**
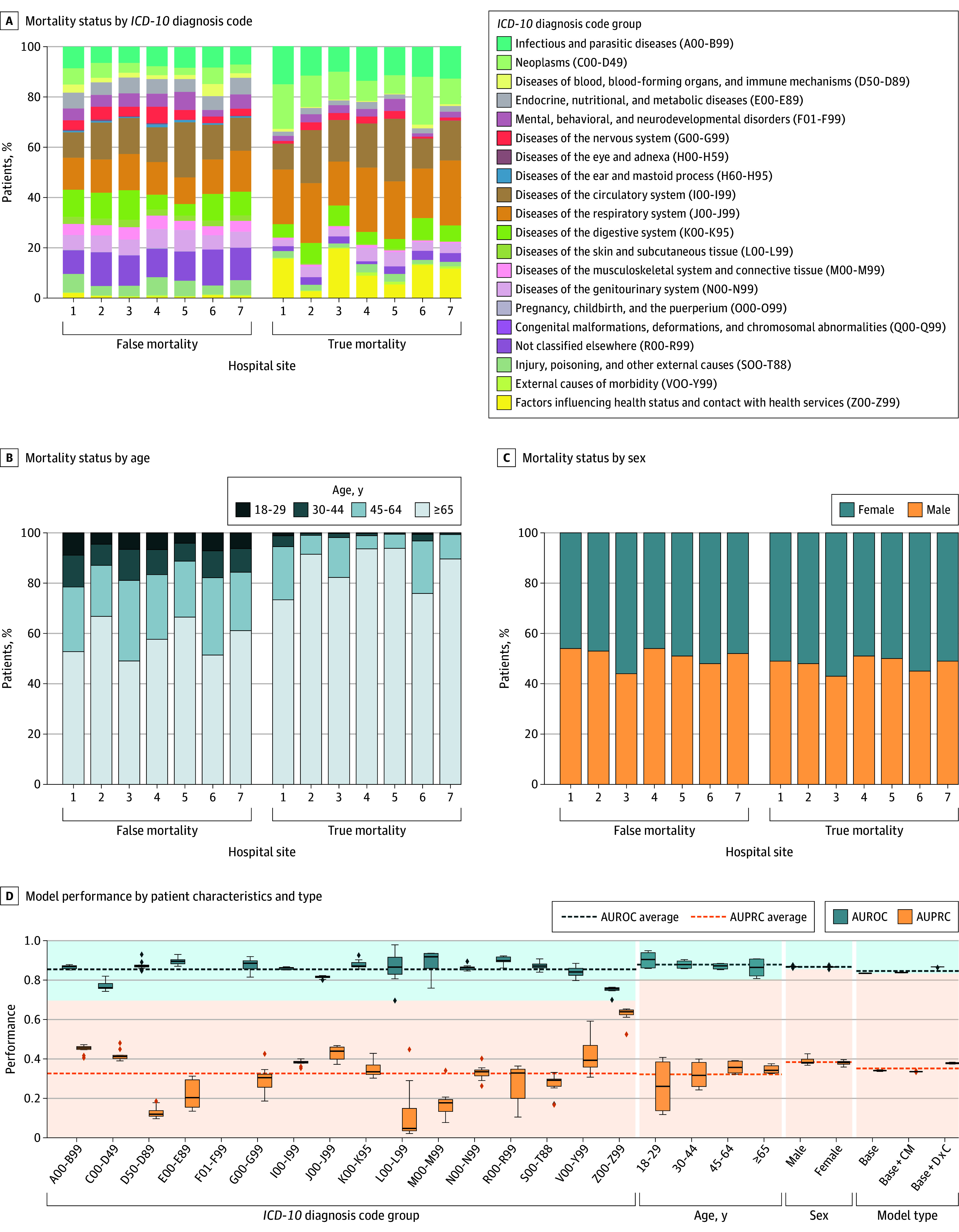
Patient Characteristics and Model Fairness Across Subgroups A, Proportion of patients by *ICD-10* diagnosis code groups across hospitals by mortality status (true or false). B, Proportion of patients by age group across hospitals by mortality status (true or false). C, Proportion of patients by sex across hospitals by mortality status (true or false). D, AUROC and AUPRC of the model without prior information (Base), with comorbidities (Base + CM), and with diagnosis codes (Base + DxC) as features. Numbers of 10 or less are suppressed to protect patient privacy. AUPRC indicates area under the precision-recall curve; AUROC, area under the receiver operating characteristic curve; and *ICD-10*, *International Statistical Classification of Diseases, Tenth Revision*.

We developed a dynamic clinical AI model to predict 14-day in-hospital mortality using EHR data (eFigures 1 and 2 and eTables 1 and 2 in Supplement 1) from 143 049 patients (mean [SD] age, 67.8 [19.6] years; 50.7% female and 49.3% male) admitted to GIM inpatient units at 7 hospitals in the Greater Toronto Area. Given demographic and diagnostic changes across hospitals (Figure 1A-C), we evaluated model fairness using AUROC and AUPRC values across *ICD-10* diagnostic groups, sex, and age. The model performed best for circulatory system diseases (AUROC for code groups I00-I99 [SD], 0.86 [0.01]; AUPRC for code groups I00-I99 [SD], 0.38 [0.01]), respiratory system diseases (AUROC for code groups J00-J99 [SD], 0.82 [0.01]; AUPRC for code groups J00-J99 [SD], 0.43 [0.04]), and infectious and parasitic diseases (AUROC for code groups A00-B99 [SD], 0.87 [0.01]; AUPRC for code groups A00-B99 [SD], 0.45 [0.02]), but it performed worse for neoplasms (AUROC for code groups C00-D49 [SD], 0.77 [0.02]; AUPRC for code groups C00-D49 [SD], 0.42 [0.03]) and health status factors (AUROC for code groups Z00-Z99 [SD], 0.75 [0.02]; AUPRC for code groups Z00-Z99 [SD], 0.63 [0.04]), which primarily consisted of patients receiving palliative care (n = 2042). Performance varied by age but remained consistent across sex (Figure 1D). Adding comorbidities maintained balanced subgroup performance, while including *ICD-10* codes improved overall performance but widened performance gaps between diagnostic groups (eFigure 3 in Supplement 1); this informed our feature selection and aligns with similar findings that report limitations using diagnosis codes directly for predictive modeling.^47^

### Data Shifts

**Figure 2.**
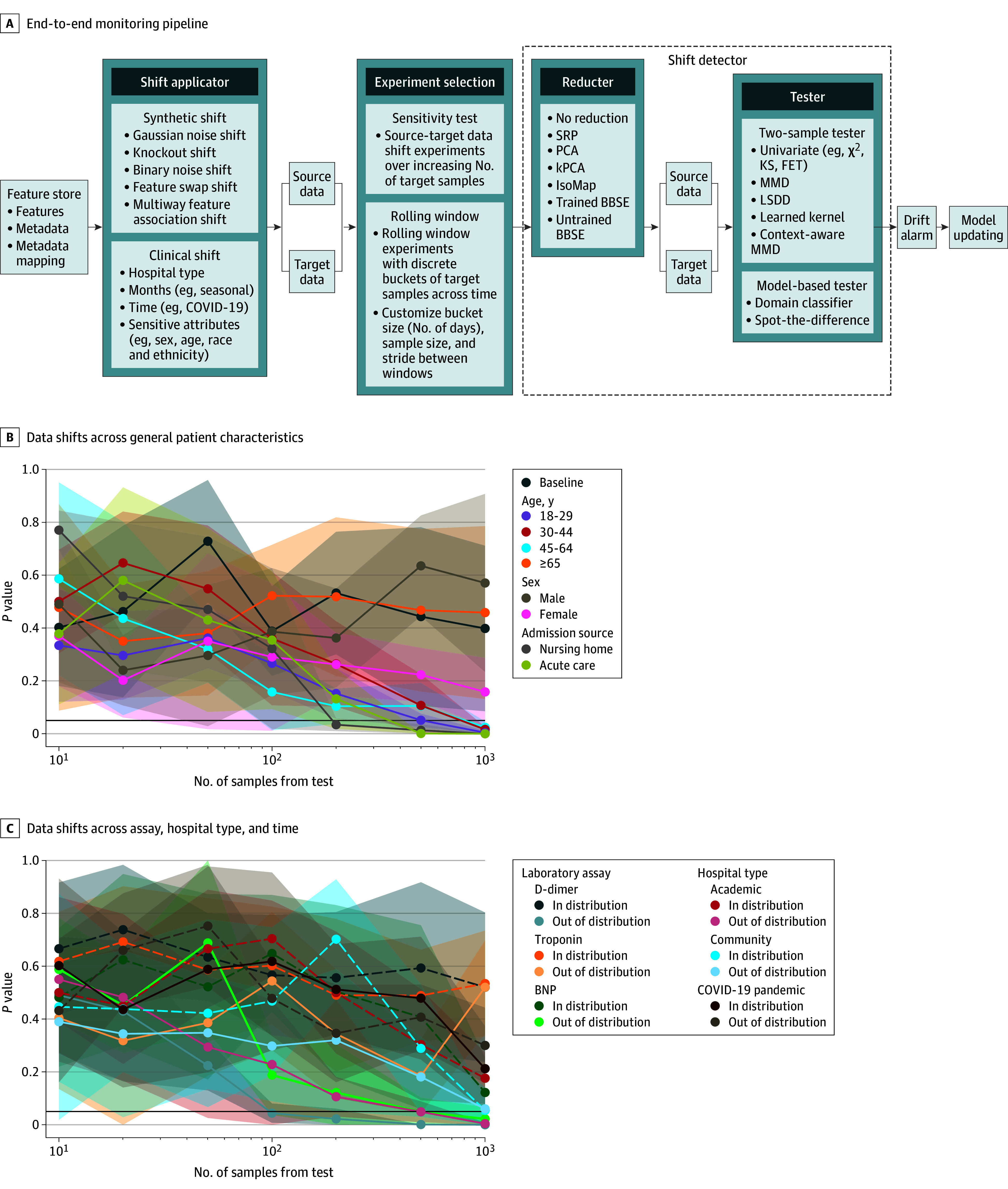
Monitoring and Evaluation of Data Shifts A, End-to-end monitoring pipeline, which processes electronic health record data through a shift applicator to generate source and target datasets, which the shift detector analyzes for drift using sensitivity tests or rolling window analysis. B, Data shifts across patient characteristics, including sex (male or female), age group (18-29, 30-44, 45-64, or ≥65 years), and admission source (acute care or nursing home). C, Data shifts due to changes in hospital type, laboratory assays (troponin, D-dimer, and brain natriuretic peptide [BNP]), and the COVID-19 pandemic. BBSE indicates black box shift estimator; FET, Fisher exact test; kPCA, kernel principal component analysis; KS, Kolmogorov-Smirnov; LSDD, least-squares density difference; MMD, maximum mean discrepancy; PCA, principal component analysis; and SRP, sparse random projections.

We detected data shifts using our monitoring pipeline (Figure 2A)^48^ across varying sample sizes for deployment threats including the COVID-19 pandemic, differences between hospital types, and changes in laboratory assays (specifically troponin, D-dimer, and BNP) (Table). We determined the optimal shift detector on synthetically induced data shifts (eTables 4-6 in Supplement 1). Age groups younger than 65 showed increasing distributional changes with decreasing age, whereas sex exhibited no significant shifts (Figure 2B). Data shifts were also observed for patients from nursing homes or acute care centers. Models transferred from academic to community hospitals but not vice versa, indicating a unidirectional hospital-type shift (Figure 2C). For critical laboratory assays, the troponin test was upgraded to high-sensitivity troponin in February 2015 at hospitals 6 and 7; however, this did not result in a data shift. At hospital 2, data shifts were observed in 2015 due to changes in the number of BNP tests and in 2018 due to changes in the number of D-dimer tests. We evaluated these shifts prospectively using a 14-day rolling window across the following periods: (1) from March 1, 2015, to July 31, 2016, to overlap the decrease in D-dimer tests; (2) from September 1, 2017, to January 31, 2019, to overlap the decrease in BNP tests; and (3) from March 1, 2019, to June 30, 2020, to overlap the start of the COVID-19 pandemic (eFigure 4 in Supplement 1). This allowed us to pinpoint the timing of the data shifts, and, in some instances, this coincided with changes in the proportion of outcomes (eFigure 5 in Supplement 1). Although no aggregate-level shift was detected for the COVID-19 pandemic, we observed shifts during April 2020, coinciding with the first wave and Ontario’s initial lockdown.

**Table.** Patient Characteristics Across Data Shifts

Characteristic	Ratio of patients from source and target				
	COVID-19 pandemic	Hospital type (academic:community)	Laboratory assay		
			Troponin	BNP	D-dimer
No. of patient encounters	20.25	2.35	8.00	7.57	8.78
Sex					
Male	21.96	2.26	8.23	7.61	8.67
Female	18.73	2.44	7.79	7.52	8.91
Age, y					
18-29	22.44	3.01	5.66	9.25	7.51
30-44	20.26	3.06	7.93	7.58	7.90
45-64	19.76	2.69	7.78	7.53	8.55
≥65	20.26	2.11	8.48	7.49	9.06
*ICD-10* diagnosis code group^a^					
Infectious and parasitic diseases (A00-B99)	33.13	5.74	9.92	8.00	8.33
Neoplasms (C00-D49)	21.88	3.87	3.25	9.00	30.00
Diseases of the blood and blood-forming organs and certain disorders involving the immune mechanism (D50-D89)	42.00	2.58	6.00	0	0
Endocrine, nutritional, and metabolic diseases (E00-E89)	46.00	4.22	5.00	3.00	4.00
Mental, behavioral, and neurodevelopmental disorders (F01-F99)	28.70	1.15	4.13	9.00	20.60
Diseases of the nervous system (G00-G99)	16.76	1.65	3.86	6.24	15.40
Diseases of the eye and adnexa (H00-H59)	78.00	3.10	3.00	3.50	5.50
Diseases of the circulatory system (I00-I99)	37.75	3.26	46.00	32.50	30.00
Diseases of the respiratory system (J00-J99)	34.36	4.35	16.33	6.32	11.90
Diseases of the digestive system (K00-K95)	9.33	5.54	4.75	7.67	14.00
Diseases of the skin and subcutaneous tissue (L00-L99)	23.60	2.91	15.00	15.00	9.00
Diseases of the musculoskeletal system and connective tissue (M00-M99)	7.00	0.75	0	1.00	1.00
Diseases of the genitourinary system (N00-N99)	20.97	2.15	9.82	9.13	7.82
Symptoms, signs, and abnormal clinical and laboratory findings, not elsewhere classified (R00-R99)	32.96	2.25	9.51	8.52	9.29
Injury, poisoning, and certain other consequences of external causes (S00-T88)	43.00	3.09	9.00	2.50	4.00
Factors influencing health status and contact with health services (Z00-Z99)	2.00	2.00	0	1.00	0
Triage level					
No information	6.03	0.67	6.12	7.00	6.56
Emergent	24.92	2.06	6.81	7.75	8.29
Nonurgent	10.37	3.72	10.00	5.00	3.00
Semiurgent	17.05	5.79	9.56	5.19	13.78
Urgent	21.55	3.36	9.87	7.18	9.41
Resuscitation	20.28	2.11	5.20	9.99	9.34
From acute care institution	17.66	2.91	6.00	7.80	6.14
From nursing home	21.60	1.33	14.40	7.02	12.36
Mean (SD) length of stay, d	1.31 (1.98)	0.85 (1.00)	0.95 (0.80)	0.88 (1.07)	1.12 (0.98)

Abbreviations: BNP, brain natriuretic peptide; *ICD-10*, *International Statistical Classification of Diseases, Tenth Revision*.

^a^ Numbers of 10 or less are suppressed to protect patient privacy.

### Performance Impact

We observed that the identified data shifts led to reduced AUROC, AUPRC, or both for the overall population (eFigure 6 in Supplement 1) and across subgroups (eFigure 7 in Supplement 1). Transferring from community to academic hospitals led to decreased model performance for patients aged 65 or older (Delta AUROC [SD], −0.05 [0.003]; Delta AUPRC [SD], −0.02 [0.01]) and to mixed results for younger patients (Delta AUROC [SD], 0.07 [0.02]; Delta AUPRC [SD], −0.26 [0.02]). The hospital-type shift may be related to the higher nursing home admissions in community hospitals (eTable 9 in Supplement 1) and urban-suburban location differences. Finally, both the BNP and D-dimer shifts resulted in changes in performance across several subgroups, particularly a decrease in AUROC (−0.12 [0.03]) and AUPRC (−0.26 [0.03]) for respiratory system diseases (J00-J99). The BNP shift also resulted in performance decreases for neurodevelopmental disorders (Delta AUROC_F01-F99_ [SD], −0.23 [0.04]; Delta AUPRC_F01-F99_ [SD], −0.19 [0.04]) and nervous system diseases (Delta AUROC_G00-G99_ [SD], −0.26 [0.04]; Delta AUPRC_G00-G99_ [SD], −0.21 [0.11]). The D-dimer shift led to decreased performance for neoplasms (Delta AUROC_C00-D49_ [SD], −0.11 [0.06]; Delta AUPRC_C00-D49_ [SD], −0.22 [0.05]), circulatory system diseases (Delta AUROC_I00-I99_ [SD], −0.09 [0.02]; Delta AUPRC_I00-I99_ [SD], −0.22 [0.05]), and digestive system diseases (Delta AUROC_K00-K95_ [SD], −0.16 [0.02]; Delta AUPRC_K00-K95_ [SD], −0.21 [0.03]).

### Cross-Site Training

**Figure 3.**
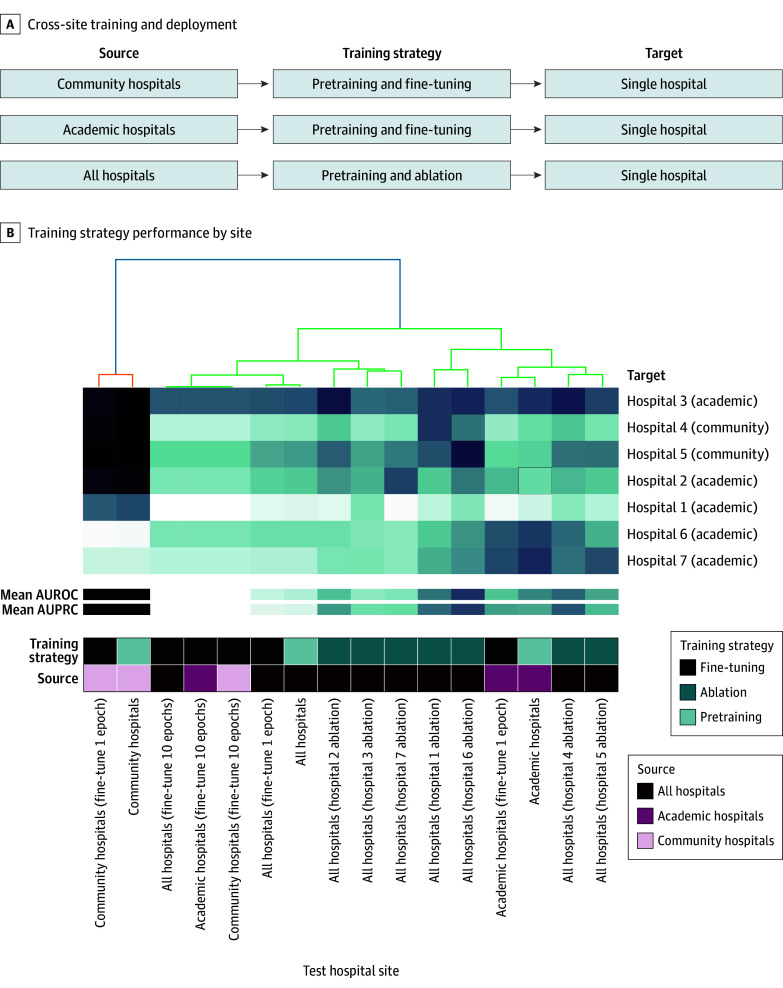
Training Strategies for Cross-Site Deployment A, Pretraining, fine-tuning, and ablation employed on a cross-site model and hospital type–specific models (community or academic). B, Heatmap of the AUROC of the training strategies across each test hospital site. *Note:* All figure icons were created for the purpose of this article. AUPRC indicates area under the precision-recall curve; AUROC, area under the receiver operating characteristic curve.

We developed AI models for 3 groups: community hospitals (hospitals 4 and 5), academic hospitals (hospitals 1, 2, 3, 6, and 7), and all hospitals combined (hospitals 1-7). We compared strategies leveraging pretraining (in which a model pretrained on source data was evaluated on out-of-distribution data from the target hospital), transfer learning (in which the model was fine-tuned on the target hospital prior to evaluation), and ablation (in which data from a single hospital were excluded prior to evaluation). Performance was assessed for each hospital using a held-out test set (Figure 3A). Training across all sites improved performance for both academic hospitals (Delta AUROC [SD], 0.04 [0.02]; Delta AUPRC [SD], 0.06 [0.04]) and community hospitals (Delta AUROC [SD], 0.04 [0.04]; Delta AUPRC [SD], 0.06 [0.02]), although community hospitals benefited most from community-specific pretraining (Delta AUROC [SD], 0.05 [0.03]; Delta AUPRC [SD], 0.06 [0.04]) (Figure 3B). Scaling multisource data by fine-tuning with increasing amounts of community hospital data consistently degraded performance on academic hospitals (eFigure 8 in Supplement 1). Hospital type–specific fine-tuning improved performance for all sites except hospital 2, where ablating hospital 3 yielded the best performance. Notably, hospitals 2 and 3 also had the largest difference in the number of individuals (17.0%) with diseases due to factors influencing health status and contact with health services—the diagnostic subgroup with the lowest performance (eFigure 7 and eTable 8 in Supplement 1).

### Data Shift–Triggered Continual Learning

**Figure 4.**
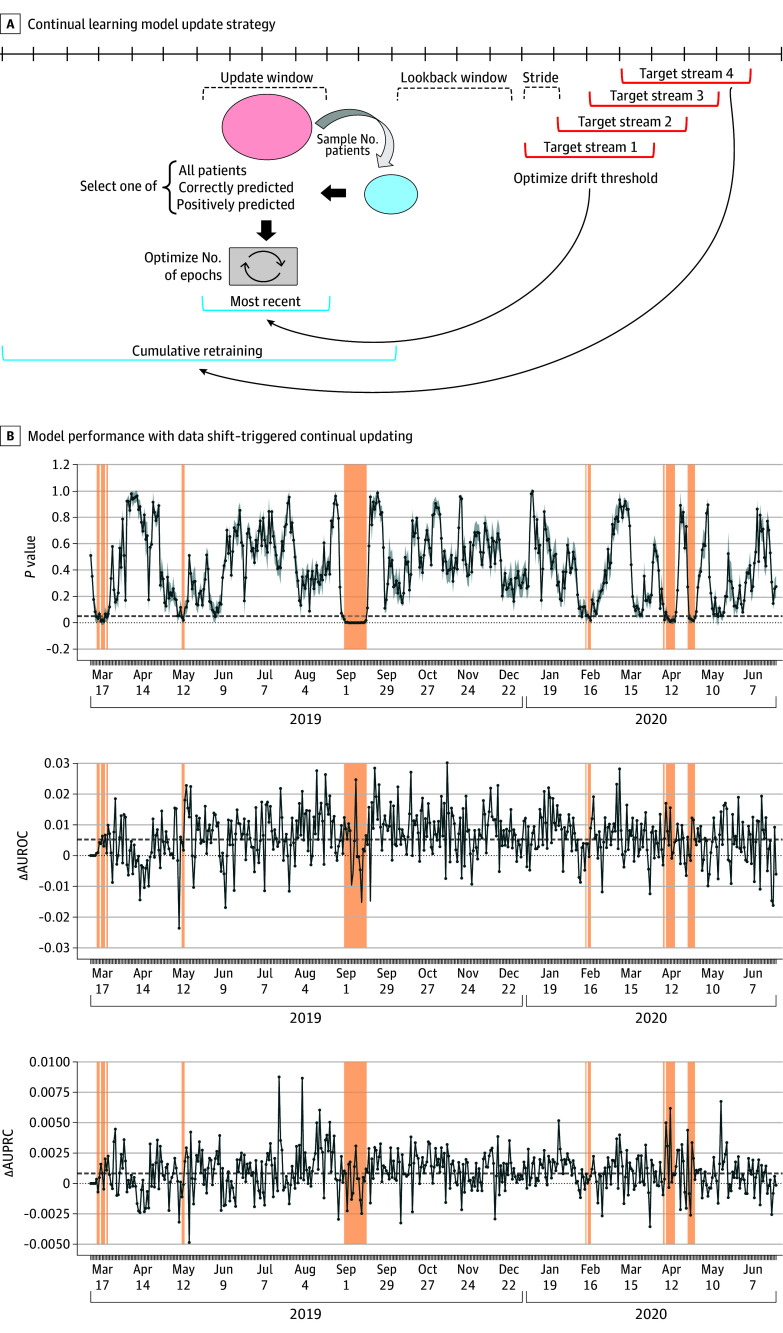
Prospective Model Updating to Mitigate Data Shifts A, Model updating strategies using target stream data from either recent number of days or cumulative encounters, with options for selective training on positive or correct predictions. Optimized parameters include data shift threshold, sample size, epochs, stride length, and window sizes. B, Clinical artificial intelligence system monitoring from March 1, 2019, to August 31, 2020, using a 14-day rolling window, with model updating triggered upon drift detection (dark shading). Performance changes in area under the receiver operating characteristic curve (ΔAUROC) and area under the precision-recall curve (ΔAUPRC) compared with the baseline model are shown, with dashed lines indicating period averages.

To mitigate model deterioration, we leveraged continual learning (Figure 4A) and found that data shift–triggered model updating improved performance compared with updating at fixed intervals (eFigure 9 in Supplement 1). The best results were obtained by updating every 120 days (eFigure 10 in Supplement 1) using data from the previous 60 days (to account for label acquisition and reporting delays) (eFigure 11 in Supplement 1), a shift detection *P* value threshold of .01 (eFigure 12 in Supplement 1), and testing with 1000 encounters (eFigure 13 in Supplement 1). Longer training periods led to catastrophic forgetting and overfitting (eFigure 14 in Supplement 1), and updating with all encounters was more effective than selectively subsampling data (eFigure 15 in Supplement 1). Overall, data shift–triggered continual updating improved model performance over time and was more effective than maintaining a locked model during the COVID-19 pandemic (Delta AUROC [SD], 0.44 [0.02]; *P* = .007, Mann-Whitney *U* test) (Figure 4B).

## Discussion

Many clinical AI systems struggle with generalizability upon deployment due to harmful data shifts that reduce performance and worsen health disparities.^49,50,51^ Current bias mitigation approaches depend on ground-truth labels, which are not always available in clinical settings.^13,52,53^ In response to calls for shared AI oversight,^54,55,56^ we introduced a proactive, label-agnostic pipeline for continuous evaluation and monitoring. This pipeline detected harmful shifts across diverse clinical scenarios, such as changes in admission sources, laboratory assays, and hospital type.

Institutional differences are a common cause of data shifts due to variations in patient demographics and data collection workflows.^10,57^ Accordingly, we detected harmful data shifts when transferring from community to academic hospitals. Transfer learning approaches leveraging models pretrained across multiple sites have been shown to improve downstream performance^30^; however, existing studies have not considered how differences in hospital type can inform training.^8,58^ Previous work suggests that more training data from certain hospitals can degrade model accuracy and compromise fairness.^59^ In our study, we found that benefits of transfer learning and scaling fine-tuned data were hospital type dependent.

Despite showing promise in medical imaging,^60^ domain generalization methods have been criticized for underperforming compared with baseline empirical risk minimization^61,62^ and exhibit limited effectiveness on clinical EHR data.^63,64^ In contrast, our approach addresses gradual shifts in sequential EHR data, aligning with prior work on temporal robustness in EHR modeling.^8,65,66^ This approach ensures timely model updates, balancing adaptation with efficiency and mitigating catastrophic forgetting. The improvements achieved by our framework can prevent misclassification of thousands of patients due to model degradation from data shifts, with impact scaling with dataset size. We also investigated key questions surrounding model updating, such as when to update a model, how much data to update, and what data to use for the update. We found that our drift-triggered continual updating strategy improved model performance and was more effective than maintaining a stale model during deployment, particularly during shifts like those seen during the COVID-19 pandemic, highlighting the potential to prevent adverse outcomes related to model degradation.

### Limitations

This study has some limitations. We present a generalizable framework for proactively evaluating clinical AI models for data shifts, enhancing robustness and deployment readiness; although the findings may be applicable to datasets with similar characteristics, they may not generalize to contexts with differing demographics or health care system factors. It is important to recognize that each prediction task, dataset, and domain is unique and, as a result, the generalizability of the specific parameters (eg, optimal drift threshold) requires optimization. Although we used established imputation methods for clinical time-series EHR data,^42,67,68^ advanced approaches like MICE (multiple imputation by chained equations) or GRU with decay warrant further exploration.^69^ Moreover, incorporation of social determinants of health could provide additional insights into fairness and generalizability across diverse populations. However, continual learning is not without risks,^70^ including catastrophic forgetting, overfitting, and feedback loops.^71,72,73^ As more data are accumulated, prospective validation is essential to confirm model adaptability and robustness in clinical environments and to understand the long-term effects of updating strategies. Moreover, the current regulatory state of continual learning systems does not clearly define how and what aspects of a clinical AI system are permitted to change following authorization.^52^

## Conclusions

Clinical AI systems are inherently complex, with unique biases and optimal updating needs. In this prognostic study, we developed a monitoring and evaluation pipeline as part of a broader machine learning operations framework for clinical AI systems^48^ to facilitate robust evaluation and monitoring prior to deployment. This approach can be integrated into clinical workflows to enhance robustness and generalizability and support data-driven decision-making by health care professionals. Too often are clinical machine learning models reported with high performance metrics without appropriate measures to evaluate biases.^53^ It is crucial to design with deployment in mind to ensure the responsible use of clinical AI systems. We aim to facilitate robust evaluation and monitoring of clinical AI systems in an effort to bridge the gaps between model development and deployment.^74,75,76^ Future research should focus on developing scalable, equitable, and efficient methods for real-time postdeployment surveillance, bias mitigation, and adaptive model updating across diverse clinical settings.

## Notes

Supplement 1. **eFigure 1.** Patient Mortality Outcome and Time to Death **eFigure 2.** Mortality Decompensation Prediction in General Internal Medicine (GIM) **eFigure 3.** Model Performance Across Diagnosis Codes **eFigure 4.** Detection of Data Shifts Over Time **eFigure 5.** Proportion of Outcomes and Mortality Events **eFigure 6.** Performance of Data Shifts **eFigure 7.** Subgroup Performance in the Presence of Harmful Data Shifts **eFigure 8.** External Data Augmentation in Cross-Site Training **eFigure 9.** Model Performance by Periodic Update Frequency **eFigure 10.** Dynamic Window Updating Performance **eFigure 11.** Impact of Lookback Window on Model Performance **eFigure 12.** Model Performance With Varying Drift Thresholds **eFigure 13.** Performance With Varying Drift Test Sample Sizes **eFigure 14.** Effect of Training Epochs on Model Performance **eFigure 15.** Model Updating Strategy Performance Comparison **eTable 1.** Electronic Health Record Features for Mortality Prediction **eTable 2.** Model Performance Metrics by Time-Series Model **eTable 3.** Hyperparameter Optimization Results **eTable 4.** Shift Detector Comparison With Gaussian Noise Varying Sample Sizes **eTable 5.** Shift Detector Comparison With Gaussian Noise Varying Noise Magnitude **eTable 6.** Shift Detector Comparison With Feature Swap **eTable 7.** Data Shift Experiment Sample Sizes **eTable 8.** Proportion of Encounters by *ICD-10* Codes and Mortality **eTable 9.** Patient Admission and Stay Characteristics by Hospital and Mortality Status
eMethods

Supplement 2.
Data Sharing Statement
