# Supplementary material for: Detecting and Remediating Harmful Data Shifts for the Responsible Deployment of Clinical AI Models
Source: JAMA Netw Open. 2025 Jun 4;8(6):e2513685. doi: 10.1001/jamanetworkopen.2025.13685 (PMC12138723; doi:10.1001/jamanetworkopen.2025.13685)
Supplement: Supplement 1. — eFigure 1. Patient Mortality Outcome and Time to Death eFigure 2. Mortality Decompensation Prediction in General Internal Medicine (GIM) eFigure 3. Model Performance Across Diagnosis Codes eFigure 4. Detection of Data Shifts Over Time eFigure 5. Proportion of Outcomes and Mortality Events eFigure 6. Performance of Data Shifts eFigure 7. Subgroup Performance in the Presence of Harmful Data Shifts eFigure 8. External Data Augmentation in Cross-Site Training eFigure 9. Model Performance by Periodic Update Frequency eFigure 10. Dynamic Window Updating Performance eFigure 11. Impact of Lookback Window on Model Performance eFigure 12. Model Performance With Varying Drift Thresholds eFigure 13. Performance With Varying Drift Test Sample Sizes eFigure 14. Effect of Training Epochs on Model Performance eFigure 15. Model Updating Strategy Performance Comparison eTable 1. Electronic Health Record Features for Mortality Prediction eTable 2. Model Performance Metrics by Time-Series Model eTable 3. Hyperparameter Optimization Results eTable 4. Shift Detector Comparison With Gaussian Noise Varying Sample Sizes eTable 5. Shift Detector Comparison With Gaussian Noise Varying Noise Magnitude eTable 6. Shift Detector Comparison With Feature Swap eTable 7. Data Shift Experiment Sample Sizes eTable 8. Proportion of Encounters by ICD-10 Codes and Mortality eTable 9. Patient Admission and Stay Characteristics by Hospital and Mortality Status eMethods [file jamanetwopen-e2513685-s001.pdf]

Supplemental Online Content

Subasri V, Krishnan A, Kore A, et al. Detecting and remediating harmful data shifts for the responsible deployment of clinical AI models. *JAMA Netw Open*. 2025;8(6):e2513685. doi:10.1001/jamanetworkopen.2025.13685

eFigure 1. Patient Mortality Outcome and Time to Death
eFigure 2. Mortality Decompensation Prediction in General Internal Medicine (GIM)
eFigure 3. Model Performance Across Diagnosis Codes
eFigure 4. Detection of Data Shifts Over Time
eFigure 5. Proportion of Outcomes and Mortality Events
eFigure 6. Performance of Data Shifts
eFigure 7. Subgroup Performance in the Presence of Harmful Data Shifts
eFigure 8. External Data Augmentation in Cross-Site Training
eFigure 9. Model Performance by Periodic Update Frequency
eFigure 10. Dynamic Window Updating Performance
eFigure 11. Impact of Lookback Window on Model Performance
eFigure 12. Model Performance With Varying Drift Thresholds
eFigure 13. Performance With Varying Drift Test Sample Sizes
eFigure 14. Effect of Training Epochs on Model Performance
eFigure 15. Model Updating Strategy Performance Comparison
eTable 1. Electronic Health Record Features for Mortality Prediction
eTable 2. Model Performance Metrics by Time-Series Model
eTable 3. Hyperparameter Optimization Results
eTable 4. Shift Detector Comparison With Gaussian Noise Varying Sample Sizes
eTable 5. Shift Detector Comparison With Gaussian Noise Varying Noise Magnitude
eTable 6. Shift Detector Comparison With Feature Swap
eTable 7. Data Shift Experiment Sample Sizes
eTable 8. Proportion of Encounters by *ICD-10* Codes and Mortality
eTable 9. Patient Admission and Stay Characteristics by Hospital and Mortality Status
eMethods

This supplemental material has been provided by the authors to give readers additional information about their work.

© 2025 Subasri V et al. *JAMA Network Open*.

eFigure 1. Patient Mortality Outcome and Time to Death

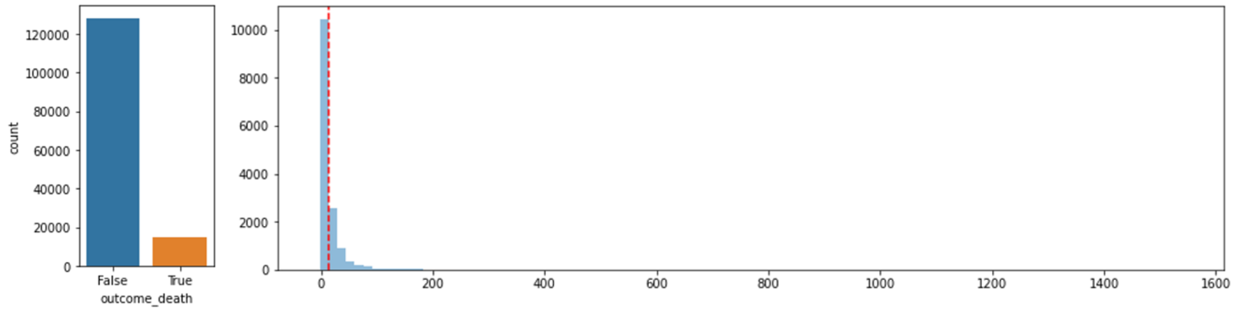

(A) Number of patients in the cohort based on the outcome death **(B)** Time to death from the time a patient is admitted for patients that experienced in-hospital mortality, dotted line indicates 2 weeks.

eFigure 2. Mortality Decompensation Prediction in General Internal Medicine (GIM)

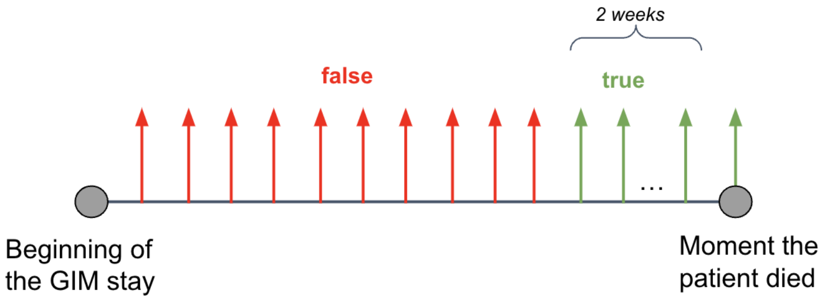

Model predicts whether patients' health deteriorates and results in hospital mortality within the next 2 weeks, every 24 hours of a GIM stay.

© 2025 Subasri V et al. *JAMA Network Open*.

eFigure 3. Model Performance Across Diagnosis Codes

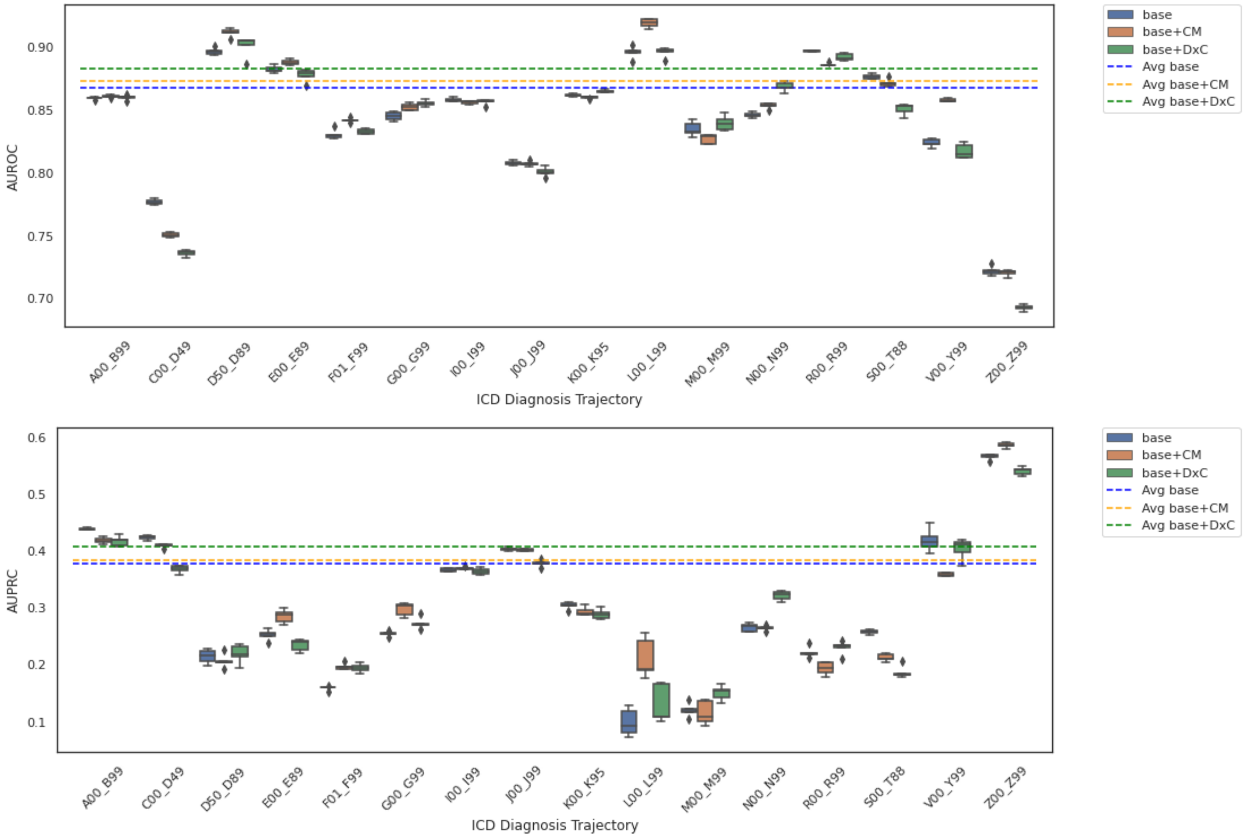

Performance of model using no prior information (base), comorbidities (base+CM) and ICD-10 diagnosis codes (base+DxC) across diagnosis codes measured using AUROC and AUPRC. The dotted line represents the average performance for each respective model, across all groups of ICD-10 diagnosis codes.

© 2025 Subasri V et al. *JAMA Network Open*.

eFigure 4. Detection of Data Shifts Over Time

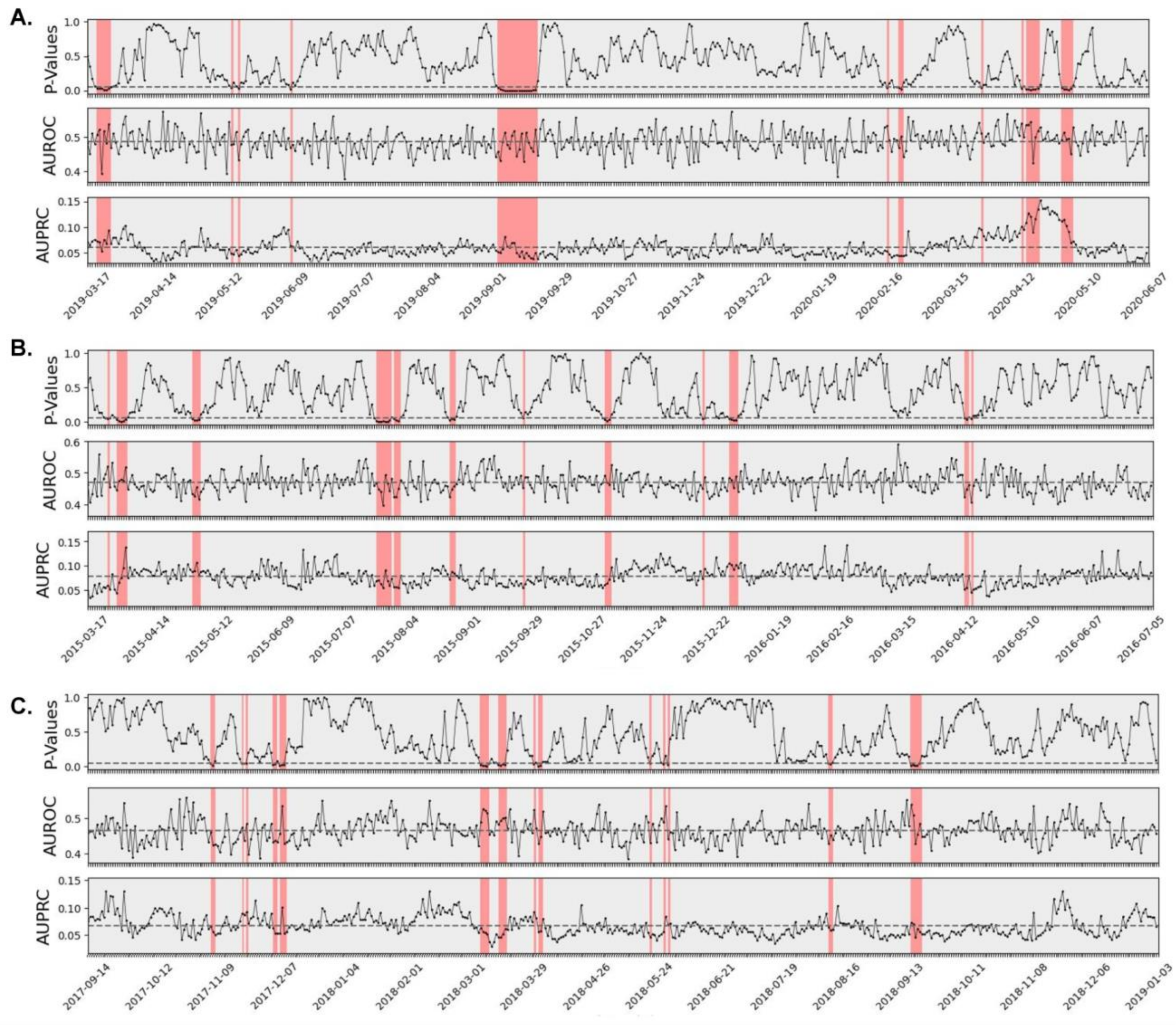

In our simulated deployment monitoring setup we used a window length of 14 days and evaluated drift every day between the data the model was trained on and the next 14 day window. Data shift tests were performed using two-sample testing of kernel mean embeddings conditioned on the model's predictions. Displayed are the p-values, AUROC and AUPRC for drift experiments for **(A)** COVID-19 **(B)** change in D-dimer tests **(C)** change in BNP tests.

eFigure 5. Proportion of Outcomes and Mortality Events

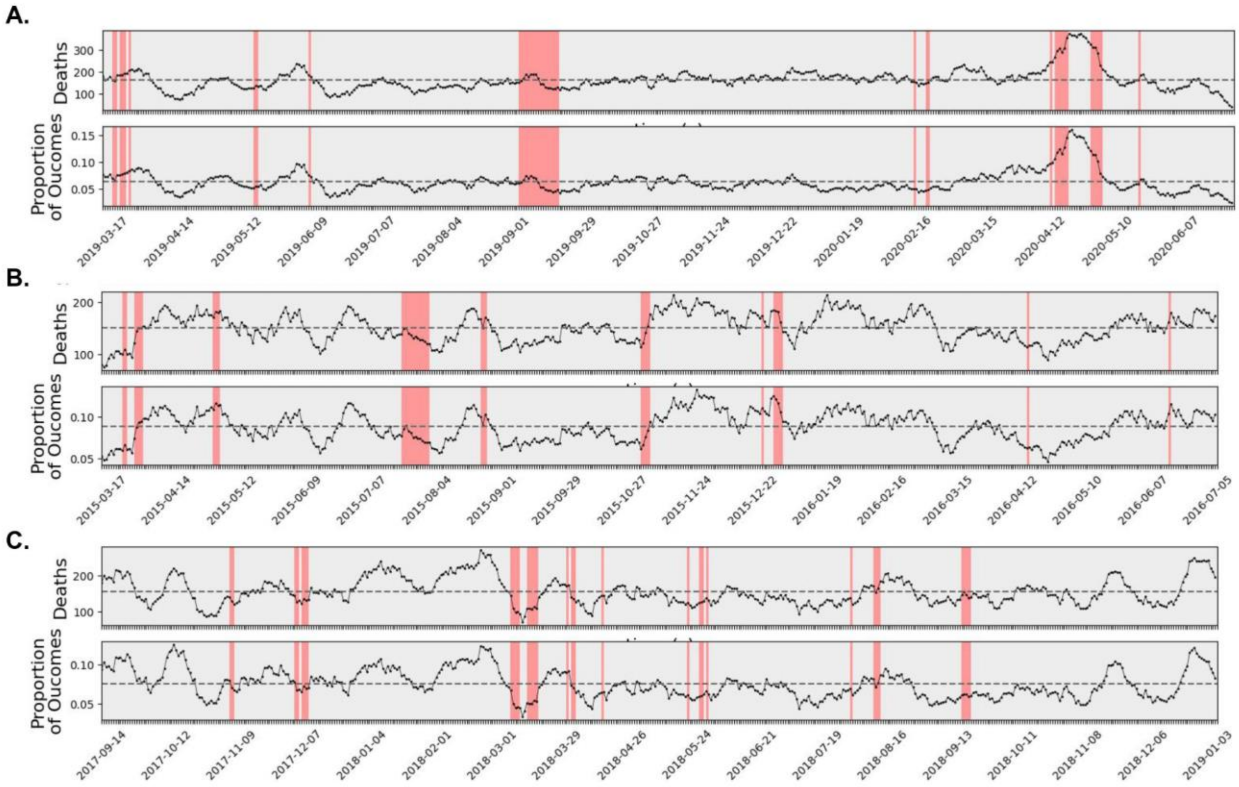

The proportion of outcomes and number of deaths using a window length of 14 days for drift experiments for **(A)** COVID-19 **(B)** change in D-dimer tests **(C)** change in BNP tests.

© 2025 Subasri V et al. *JAMA Network Open*.

eFigure 6. Performance of Data Shifts

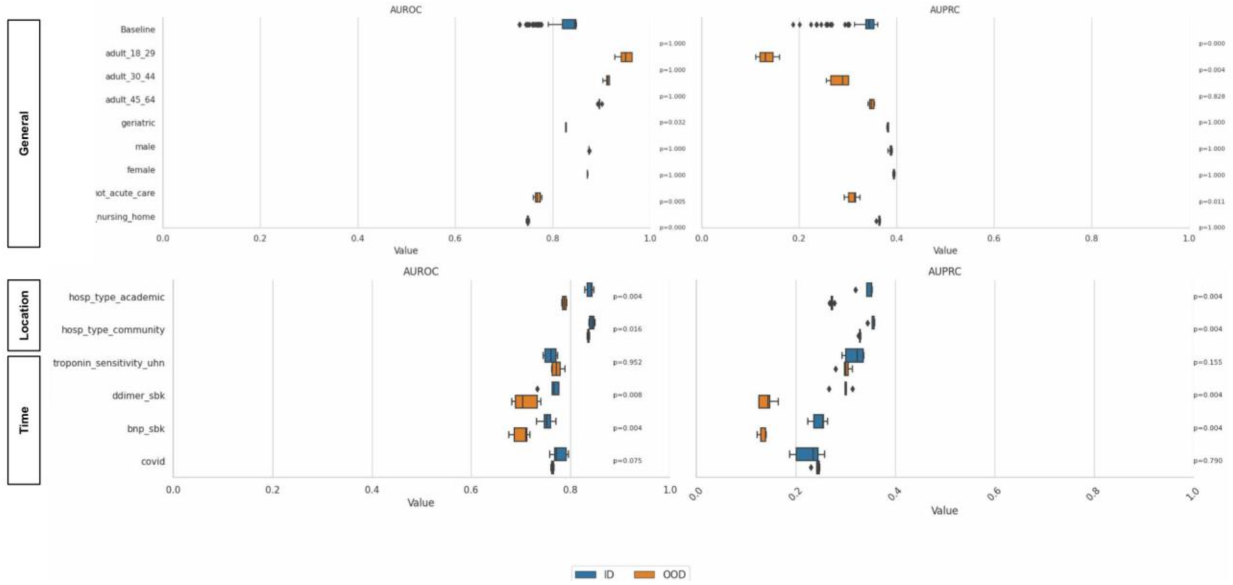

Comparison of AUROC and AUPRC between baseline (ID) and out-of-distribution (OOD) scenarios using one-sided Mann-Whitney U tests.

© 2025 Subasri V et al. *JAMA Network Open*.

eFigure 7. Subgroup Performance in the Presence of Harmful Data Shifts

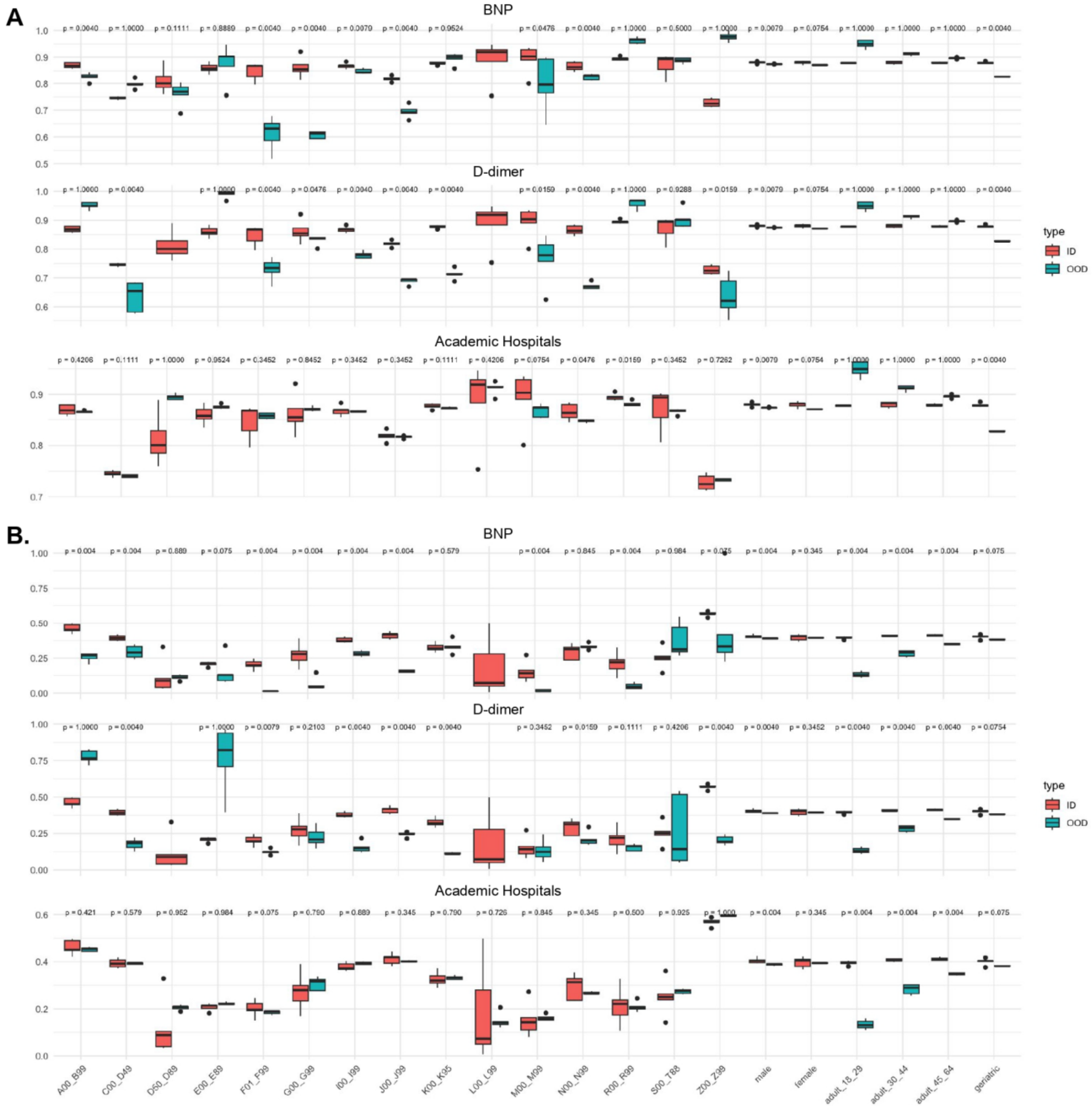

(A) AUROC and **(B)** AUPRC of in-distribution (ID) and out-of-distribution (OOD) data across ICD-10 diagnosis codes, age and sex, for scenarios where harmful data shifts were detected due to changes in lab assays: BNP and D-dimer, and transfer from community to academic hospitals. P-values were calculated using a one-sided Mann-Whitney U test.

© 2025 Subasri V et al. *JAMA Network Open*.

eFigure 8. External Data Augmentation in Cross-Site Training

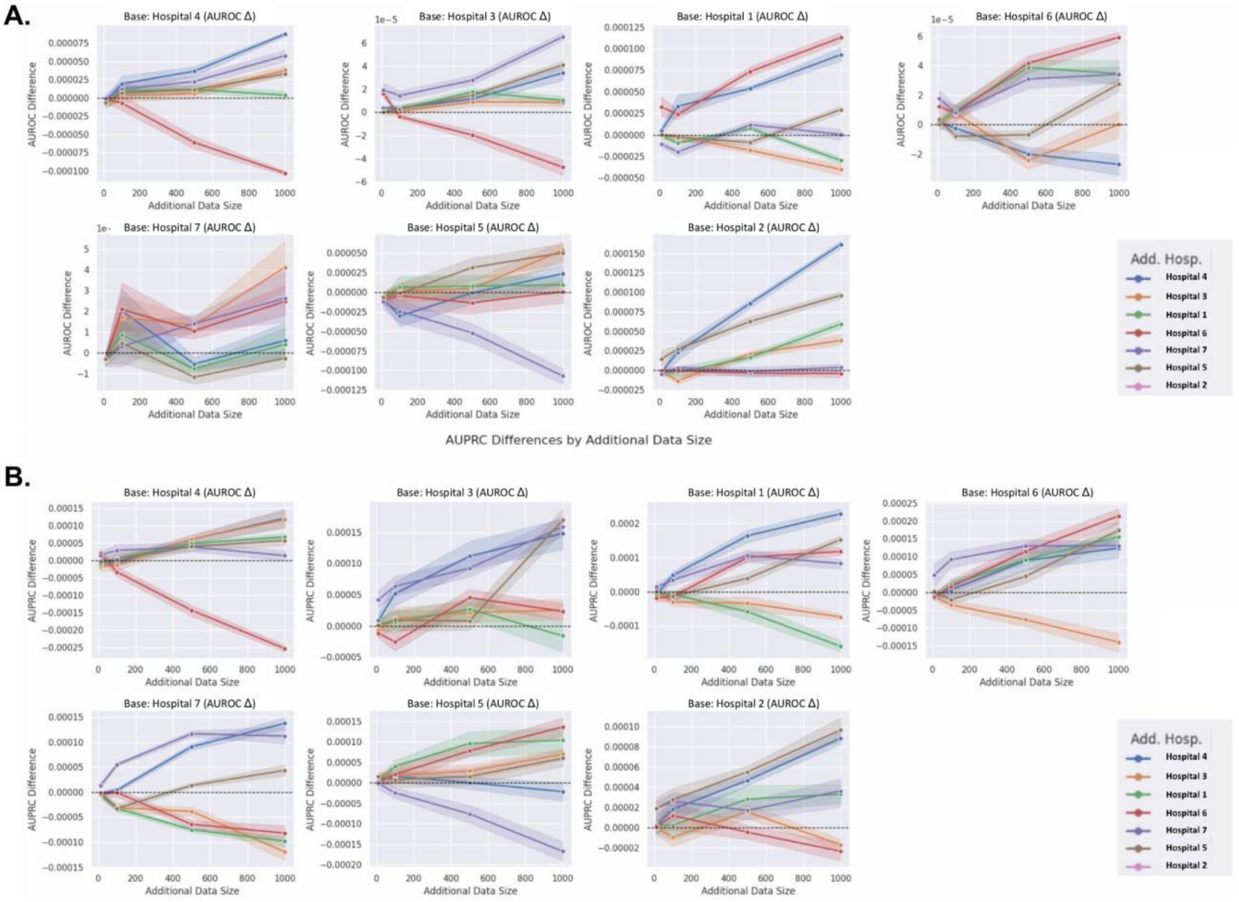

When evaluating performance on a base hospital's test set, we measured the impact of incrementally adding training data from external hospitals on performance measured by **(A)** AUROC and **(B)** AUPRC.

eFigure 9. Model Performance by Periodic Update Frequency

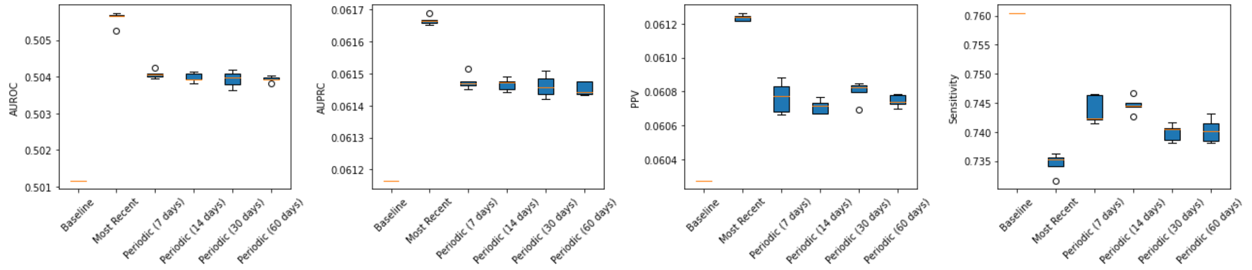

Comparison of AUROC, AUPRC, PPV, and sensitivity when updating periodically every n = 7, 14, 30, and 60 days.

© 2025 Subasri V et al. *JAMA Network Open*.

eFigure 10. Dynamic Window Updating Performance

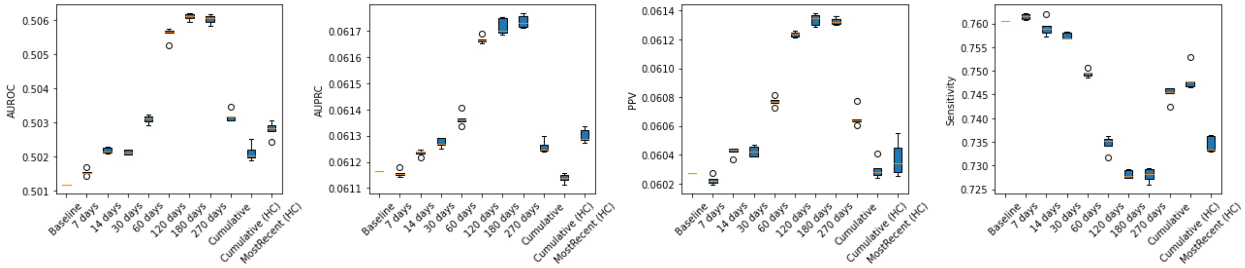

Comparison of AUROC, AUPRC, PPV, and sensitivity across strategies updating using a dynamic window of the most recent encounters (n = 7, 14, 30, 60, 120, 180, 270 days) and cumulatively.

eFigure 11. Impact of Lookback Window on Model Performance

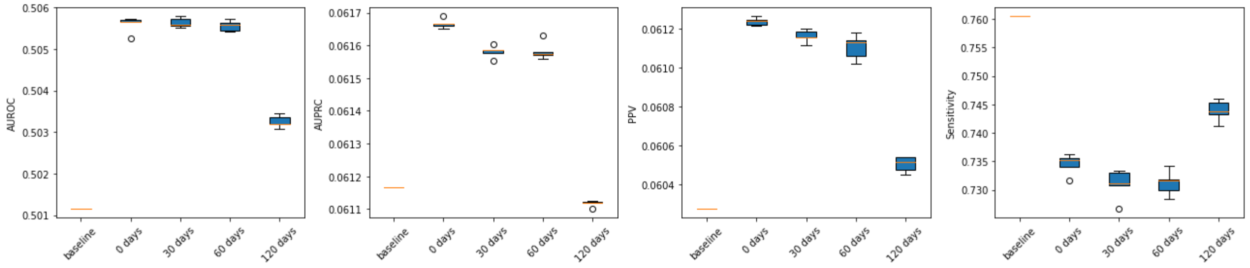

Comparison of AUROC, AUPRC, PPV, and sensitivity across increasing lookback windows (n = 0, 30, 60, 120 days).

eFigure 12. Model Performance With Varying Drift Thresholds

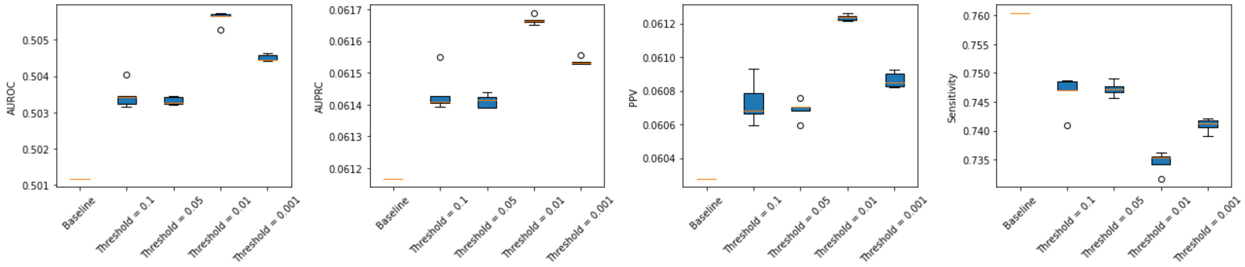

Comparison of AUROC, AUPRC, PPV, and sensitivity across varying drift p-value thresholds for model updating (p = 0.1, 0.05, 0.01, 0.001).

eFigure 13. Performance With Varying Drift Test Sample Sizes

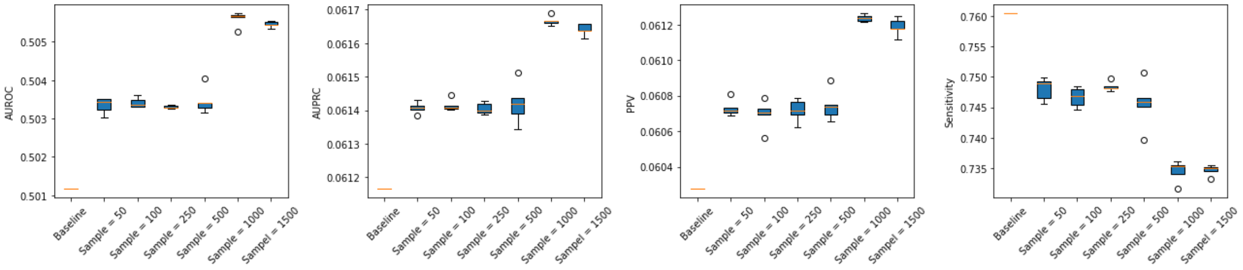

Comparison of AUROC, AUPRC, PPV, and sensitivity across varying sample sizes for drift tests (n = 50, 100, 250, 500, 1000, 1500).

eFigure 14. Effect of Training Epochs on Model Performance

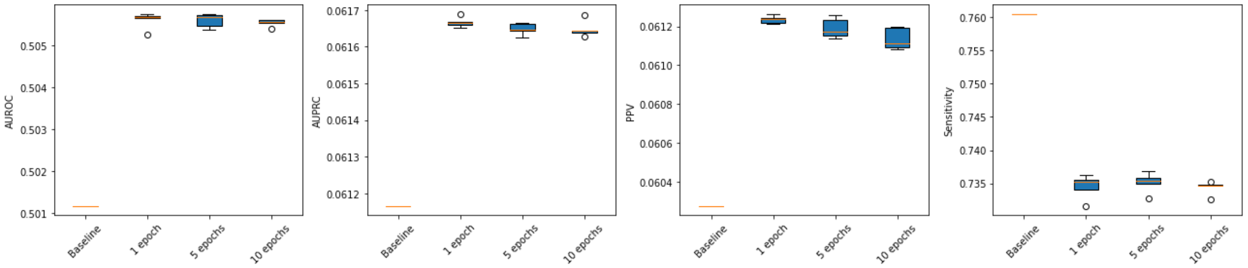

Comparison of AUROC, AUPRC, PPV, and sensitivity across varying epochs for model updating (n = 1, 5, 10).

eFigure 15. Model Updating Strategy Performance Comparison

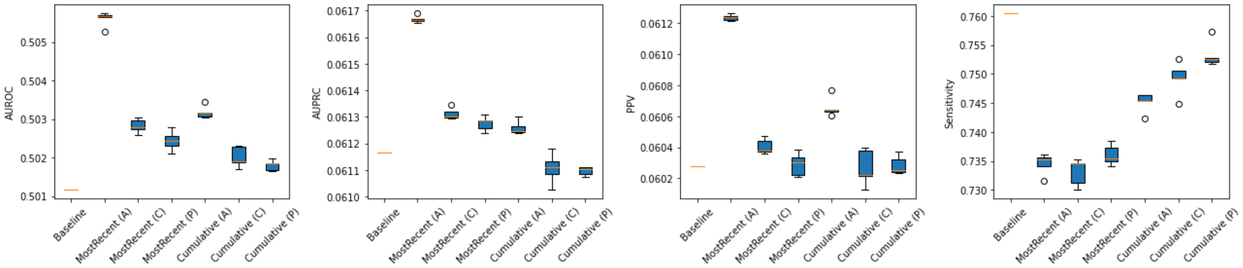

Comparison of AUROC, AUPRC, PPV, and sensitivity when updating using the most recent and cumulative updating strategy with all encounters (A), correctly predicted encounters (C) or positively predicted encounters (P).

© 2025 Subasri V et al. *JAMA Network Open*.

eTable 1. Electronic Health Record Features for Mortality Prediction

Feature Type	# of Features	Features
Administrative	16	sex, age, prev_encounter_count, triage_level_emergent, triage_level_no_info, triage_level_non-urgent, triage_level_resuscitation, triage_level_semi-urgent, triage_level_urgent, readmission_new_to_acute, readmission_nota, readmission_planned_from_acute, readmission_unplanned_7_day_acute, readmission_unplanned_7_day_day_surg, readmission_unplanned_8_to_28_day_acute, from_nursing_home_mapped, from_acute_care_institution_mapped
Interventions	6	unmapped_intervention, inv_mech_vent_mapped, endoscopy_mapped, dialysis_mapped, surgery_mapped, interventional
Labs	55	albumin, alp, alt, aptt, arterial paco2, arterial pao2, arterial ph, ast, bicarbonate, bilirubin, blood urea nitrogen, calcium, calcium ionized, creatinine, crp, d-dimer, esr, ferritin, fibrinogen, glucose fasting, glucose point of care, glucose random, hba1c, hematocrit, hemoglobin, high sensitivity troponin, influenza, inr, ketone, lactate arterial, lactate venous, ldh, lipase, lymphocyte, mean cell volume, neutrophils, other, platelet count, potassium, pt, serum alcohol, serum osmolality, sodium, troponin, tsh, urinalysis, urine osmolality, urine sodium, urine specific gravity, venous pco2, venous ph, vitamin b12, vitamin d, white blood cell count
Imaging Reports	5	ct, mri, x-ray, echo, ultrasound
Blood Transfusions	2	rbc, non-rbc
Comorbidities<br><br>*(Only used in Base+CM)*	18	Kidney disease: N18, N19<br>Ischemic heart disease: I20-I52<br>Cerebrovascular disease: I60-69<br>Hypertension: I10-I15<br>Diabetes: E10-E13<br>Hyperlipidemia: E78<br>Hypertension: I10<br>Congestive heart failure: I50<br>Cancer: C00-D49<br>Dyspnea: R06<br>COPD: J44<br>Asthma: J45<br>Pulmonary embolism: I26<br>Connective tissue disease: I30-I36<br>Inflammatory bowel disease: K50, K51,<br>Osteoarthritis: M15-M19<br>Rheumatoid arthritis: M05-M14<br>HIV: B20-B24

© 2025 Subasri V et al. *JAMA Network Open*.

Feature Type	# of Features	Features
ICD-10 Diagnosis Codes<br><br>*(Only used in Base+DxC)*	22	Certain infectious and parasitic diseases: A00-B99<br>Neoplasms: C00-D49<br>Diseases of the blood and blood-forming organs and certain disorders involving the immune mechanism: D50-D89<br>Endocrine, nutritional and metabolic diseases: E00-E89<br>Mental, Behavioral and Neurodevelopmental disorders: F01-F99<br>Diseases of the nervous system: G00-G99<br>Diseases of the eye and adnexa: H00-H59<br>Diseases of the ear and mastoid process: H60-H95<br>Diseases of the circulatory system: I00-I99<br>Diseases of the respiratory system: J00-J99<br>Diseases of the digestive system: K00-K95<br>Diseases of the skin and subcutaneous tissue: L00-L99<br>Diseases of the musculoskeletal system and connective tissue: M00-M99<br>Diseases of the genitourinary system: N00-N99<br>Pregnancy, childbirth and the puerperium: O00-O99<br>Certain conditions originating in the perinatal period: P00-P96<br>Congenital malformations, deformations and chromosomal abnormalities: Q00-Q99<br>Symptoms, signs and abnormal clinical and laboratory findings, not elsewhere classified: R00-R99<br>Injury, poisoning and certain other consequences of external causes: S00-T88<br>External causes of morbidity: V00-Y99<br>COVID19: U07-U08<br>Factors influencing health status and contact with health services: Z00-Z99

eTable 2. Model Performance Metrics by Time Series Model

Model	AUROC	AUPRC	Sensitivity	PPV
Recurrent neural network (RNN)	0.835 ± 0.0005	0.328 ± 0.0026	0.887 ± 0.0030	0.162 ± 0.0016
Gated recurrent unit (GRU)	0.838 ± 0.0013	0.344 ± 0.0030	0.865 ± 0.0067	0.177 ± 0.0008
Long short-term memory (LSTM)	0.846 ± 0.0009	0.349 ± 0.0022	0.887 ± 0.0045	0.173 ± 0.0013

AUROC, AUPRC, sensitivity, and PPV of the test set, for time series models including RNN, GRU and LSTM models.

© 2025 Subasri V et al. *JAMA Network Open*.

eTable 3. Hyperparameter Optimization Results

Parameter	Parameter Value	AUROC	AUPRC
Hidden cells	32	0.682+0.129	0.167+0.085
	64	0.694+0.123	0.175+0.086
	128	0.707+0.112	0.184+0.081
Layers	2	0.745 ± 0.106	0.201 ± 0.085
	4	0.684 ± 0.122	0.168 ± 0.082
	6	0.654 ± 0.121	0.157 ± 0.080
Dropout	0.1	0.695 ± 0.084	0.175 ± 0.121
	0.2	0.694 ± 0.084	0.176 ± 0.122
	0.3	0.694 ± 0.085	0.176 ± 0.122
Learning rate	0.000001	0.586 ± 0.041	0.085 ± 0.019
	0.0001	0.802 ± 0.107	0.263 ± 0.058
	0.01	0.695 ± 0.092	0.178 ± 0.043
Number of epochs	128	0.695 ± 0.124	0.176 ± 0.085
	256	0.694 ± 0.122	0.175 ± 0.084
	348	0.693 ± 0.121	0.175 ± 0.084
Weight decay	0.0000001	0.720 ± 0.108	0.185 ± 0.085
	0.00001	0.717 ± 0.109	0.185 ± 0.086
	0.001	0.646 ± 0.133	0.156 ± 0.079

Hyperparameter optimization was performed across the number of hidden cells {32, 64, 128}, number of layers {2, 4, 6}, dropout, {0.1, 0.2, 0.3}, learning rate {$1x10^{-6}$, $1x10^{-4}$, $1x10^{-2}$}, number of epochs {128, 256, 348}, weight decay {$1x10^{-7}$, $1x10^{-5}$, $1x10^{-3}$}. Average AUROC and AUPRC across all parameters are reported.

© 2025 Subasri V et al. *JAMA Network Open*.

eTable 4. Shift Detector Comparison With Gaussian Noise Varying Sample Sizes

Tester	Reduction	num_samples=0.25	num_samples=0.5	num_samples=1.0
**Univariate (KS)**	**No reduction**	[0.5, 0.5, 0.5, 0.5, 0.5, 0.5, 0.5]	[0.5, 0.5, 0.5, 0.5, 0.5, 0.5, 0.5]	[0.5, 0.5, 0.5, 0.5, 0.5, 0.5, 0.5]
	**PCA**	[0.53, 0.53, 0.53, 0.52, 0.53, 0.51, 0.51]	[0.51, 0.52, 0.52, 0.53, 0.51, 0.5, 0.5]	[0.54, 0.51, 0.52, 0.52, 0.51, 0.5, 0.5]
	**SRP**	[0.53, 0.53, 0.51, 0.5, 0.5, 0.5, 0.5]	[0.52, 0.53, 0.5, 0.5, 0.5, 0.5, 0.5]	[0.54, 0.5, 0.5, 0.5, 0.5, 0.5, 0.5]
	**kPCA (poly)**	[0.5, 0.5, 0.5, 0.5, 0.5, 0.5, 0.5]	[0.56, 0.54, 0.55, 0.53, 0.5, 0.5, 0.5]	[0.56, 0.55, 0.54, 0.53, 0.52, 0.5, 0.5]
	**BBSEs**	[0.52, 0.6, 0.68, 0.78, 0.88, 0.9, 0.92]	[0.6, 0.66, 0.68, 0.77, 0.85, 0.92, 0.88]	[0.56, 0.6, 0.69, 0.76, 0.8, 0.91, 0.91]
	**Classifier**	[0.5, 0.5, 0.5, 0.5, 0.5, 0.5, 0.5]	[0.5, 0.5, 0.5, 0.5, 0.5, 0.5, 0.5]	[0.5, 0.5, 0.5, 0.5, 0.5, 0.5, 0.5]
	**Spot-the-diff**	[0.87, 0.97, 0.98, 0.98, 0.98, 0.96, 0.94]	[0.88, 0.98, 0.98, 0.98, 0.98, 0.98, 0.97]	[0.88, 0.94, 0.98, 0.98, 0.96, 0.96, 0.98]
**Chi-squared**	**BBSEh**	[0.5, 0.5, 0.5, 0.5, 0.5, 0.5, 0.5]	[0.5, 0.5, 0.5, 0.5, 0.5, 0.5, 0.5]	[0.5, 0.5, 0.5, 0.5, 0.5, 0.5, 0.5]
**Multivariate (MMD)**	**No reduction**	[0.97, 0.98, 0.96, 0.98, 0.98, 0.98, 0.99]	[0.94, 0.98, 0.98, 0.96, 0.99, 0.94, 0.96]	[0.97, 0.97, 0.98, 0.99, 0.96, 0.96, 0.88]
	**PCA**	[0.52, 0.55, 0.69, 0.88, 0.96, 0.97, 0.97]	[0.51, 0.59, 0.75, 0.9, 0.96, 0.96, 0.84]	[0.54, 0.6, 0.81, 0.93, 0.98, 0.96, 0.93]
	**SRP**	[0.98, 0.99, 0.98, 0.98, 0.96, 0.94, 0.92]	[0.98, 0.97, 0.99, 0.96, 0.98, 0.96, 0.98]	[0.98, 0.98, 1.0, 0.98, 0.98, 0.96, 0.92]
	**kPCA (poly)**	[0.48, 0.49, 0.48, 0.49, 0.52, 0.46, 0.46]	[0.47, 0.51, 0.52, 0.5, 0.58, 0.73, 0.84]	[0.54, 0.5, 0.58, 0.57, 0.62, 0.82, 0.92]
	**BBSEs**	**[0.48, 0.5, 0.58, 0.88, 0.96, 0.96, 0.96]**	**[0.5, 0.5, 0.66, 0.92, 0.98, 0.98, 0.99]**	**[0.52, 0.51, 0.69, 0.94, 0.98, 0.98, 0.98]**

Data shift detector comparison in the presence of synthetic data shifts due to gaussian noise (noise_amt=1.0) for increasing sample size n= {0.25, 0.5, 1.0}. The top performing shift detection method is bolded.

© 2025 Subasri V et al. *JAMA Network Open*.

eTable 5. Shift Detector Comparison With Gaussian Noise Varying Noise Magnitude

Tester	Reduction	noise_amt=0.1	noise_amt=1.0	noise_amt=10.0
**Univariate (KS)**	**None**	[0.5, 0.5, 0.5, 0.5, 0.5, 0.5, 0.5]	[0.5, 0.5, 0.5, 0.5, 0.5, 0.5, 0.5]	[0.5, 0.5, 0.5, 0.5, 0.5, 0.5, 0.5]
	**PCA**	[0.5, 0.52, 0.51, 0.5, 0.51, 0.5, 0.51]	[0.52, 0.52, 0.53, 0.51, 0.52, 0.51, 0.5]	[0.52, 0.53, 0.52, 0.52, 0.5, 0.5, 0.5]
	**SRP**	[0.48, 0.48, 0.5, 0.49, 0.5, 0.5, 0.5]	[0.56, 0.52, 0.51, 0.5, 0.5, 0.5, 0.5]	[0.54, 0.52, 0.5, 0.5, 0.5, 0.5, 0.5]
	**kPCA(poly)**	[0.51, 0.52, 0.48, 0.48, 0.48, 0.5, 0.5]	[0.53, 0.54, 0.52, 0.53, 0.52, 0.52, 0.5]	[0.54, 0.55, 0.52, 0.52, 0.5, 0.5, 0.5]
	**BBSEs**	[0.49, 0.44, 0.5, 0.5, 0.5, 0.48, 0.5]	[0.52, 0.61, 0.61, 0.68, 0.68, 0.77, 0.81]	[0.88, 0.92, 0.92, 0.91, 0.85, 0.82, 0.75]
	**Classifier**	[0.5, 0.5, 0.5, 0.5, 0.5, 0.5, 0.5]	[0.5, 0.5, 0.5, 0.5, 0.5, 0.5, 0.5]	[0.5, 0.5, 0.5, 0.5, 0.5, 0.5, 0.5]
	**Spot-the-diff**	[0.51, 0.51, 0.5, 0.48, 0.5, 0.53, 0.54]	[0.86, 0.98, 0.96, 0.98, 0.97, 0.98, 1.0]	[1.0, 0.98, 0.97, 0.98, 0.98, 0.99, 0.98]
**Chi-squared**	**BBSEh**	[0.5, 0.5, 0.5, 0.5, 0.5, 0.5, 0.5]	[0.5, 0.5, 0.5, 0.5, 0.5, 0.5, 0.5]	[0.5, 0.5, 0.5, 0.5, 0.5, 0.5, 0.5]
**Multivariate (MMD)**	**No reduction**	[0.48, 0.5, 0.5, 0.51, 0.52, 0.57, 0.64]	[0.96, 1.0, 0.99, 0.97, 0.97, 0.96, 0.85]	[0.99, 0.96, 0.97, 0.98, 1.0, 0.98, 1.0]
	**PCA**	[0.5, 0.48, 0.49, 0.52, 0.5, 0.5, 0.48]	[0.51, 0.56, 0.66, 0.84, 0.96, 0.99, 0.99]	[0.98, 0.96, 0.99, 0.96, 0.98, 0.96, 0.98]
	**SRP**	[0.5, 0.5, 0.5, 0.53, 0.5, 0.55, 0.58]	[0.98, 0.97, 0.98, 0.97, 0.96, 0.96, 0.97]	[1.0, 0.98, 0.98, 0.98, 0.97, 0.98, 1.0]
	**kPCA(poly)**	[0.52, 0.5, 0.52, 0.5, 0.46, 0.44, 0.31]	[0.5, 0.49, 0.5, 0.53, 0.6, 0.72, 0.84]	[0.96, 0.97, 0.94, 0.93, 0.86, 0.76, 0.62]
	**BBSEs**	**[0.52, 0.51, 0.51, 0.5, 0.53, 0.54, 0.54]**	**[0.5, 0.52, 0.62, 0.9, 0.96, 0.94, 0.96]**	**[0.78, 0.96, 0.99, 0.99, 0.98, 1.0, 0.98]**

Data shift detector comparison in the presence of synthetic data shifts due to gaussian noise for increasing noise magnitude, noise_amt= {0.25, 0.5, 1.0}. The top performing shift detection method is bolded.

© 2025 Subasri V et al. *JAMA Network Open*.

eTable 6. Shift Detector Comparison With Feature Swap

Tester	Reduction	n_shuffle=0.25	n_shuffle=0.75
**Univariate (KS)**	**None**	[0.50, 0.50, 0.50, 0.50, 0.50, 0.50, 0.50]	[0.50, 0.50, 0.50, 0.50, 0.50, 0.50, 0.50]
	**PCA**	[0.51, 0.5, 0.5, 0.5, 0.5, 0.48, 0.5]	[0.52, 0.48, 0.52, 0.5, 0.51, 0.51, 0.5]
	**SRP**	[0.5, 0.48, 0.48, 0.46, 0.48, 0.5, 0.5]	[0.49, 0.49, 0.51, 0.5, 0.5, 0.5, 0.5]
	**kPCA(poly)**	[0.46, 0.46, 0.48, 0.47, 0.49, 0.49, 0.5]	[0.5, 0.49, 0.49, 0.46, 0.5, 0.49, 0.5]
	**BBSEs**	[0.47, 0.5, 0.44, 0.41, 0.32, 0.36, 0.32]	[0.48, 0.5, 0.42, 0.43, 0.38, 0.36, 0.29]
	**Classifier**	[0.5, 0.5, 0.5, 0.5, 0.5, 0.5, 0.5]	[0.5, 0.5, 0.5, 0.5, 0.5, 0.5, 0.5]
	**Spot-the-diff**	[0.52, 0.48, 0.5, 0.52, 0.52, 0.52, 0.52]	[0.5, 0.53, 0.48, 0.48, 0.5, 0.48, 0.48]
**Chi-squared**	**BBSEh**	[0.50, 0.50, 0.50, 0.50, 0.50, 0.50, 0.50]	[0.50, 0.50, 0.50, 0.50, 0.50, 0.50, 0.50]
**Multivariate (MMD)**	**None**	[0.5, 0.52, 0.51, 0.5, 0.48, 0.49, 0.48]	[0.49, 0.5, 0.5, 0.48, 0.52, 0.54, 0.52]
	**PCA**	[0.49, 0.51, 0.5, 0.52, 0.5, 0.5, 0.52]	[0.49, 0.52, 0.5, 0.54, 0.54, 0.58, 0.64]
	**SRP**	[0.48, 0.52, 0.5, 0.48, 0.47, 0.46, 0.44]	[0.48, 0.52, 0.52, 0.47, 0.53, 0.5, 0.5]
	**kPCA(poly)**	[0.49, 0.48, 0.48, 0.5, 0.46, 0.49, 0.46]	[0.51, 0.48, 0.48, 0.48, 0.5, 0.44, 0.36]
	**BBSEs**	**[0.5, 0.5, 0.51, 0.48, 0.5, 0.5, 0.52]**	**[0.48, 0.48, 0.51, 0.54, 0.52, 0.64, 0.76]**

Data shift detector comparison in the presence of synthetic data shifts due to feature swap of 25% and 75% of the features across increasing sample sizes. The top performing shift detection method is bolded.

© 2025 Subasri V et al. *JAMA Network Open*.

eTable 7. Data Shift Experiment Sample Sizes

Experiment	Shift Type	Source (Training)		Source (Test)		Target (Test)	
		Positive	Negative	Positive	Negative	Positive	Negative
Male	General	24258	272916	6245	100685	19530	241189
Female	General	24258	272916	6245	100685	20940	234340
Adult (18-29)	General	24258	272916	6245	100685	90	15904
Adult (30-44)	General	24258	272916	6245	100685	488	29230
Adult (45-64)	General	24258	272916	6245	100685	4202	96711
Adult (65+)	General	24258	272916	6245	100685	5814	167034
From acute care	General	24258	272916	6245	100685	580	5223
From nursing home	General	24258	272916	6245	100685	8111	47404
COVID-19	Time	3397	57607	922	14480	1571	21986
Troponin	Time	3226	30341	852	7607	5077	62561
D-dimer	Time	2251	29169	629	48548	2451	7322
BNP	Time	3334	47660	931	11798	526	10219
Community	Location	22046	260251	5210	64905	13214	150373
Academic	Location	10551	120144	2663	30229	27256	325156

Training and test sample sizes used for data shift experiments.

eTable 8. Proportion of Encounters by *ICD-10* Codes and Mortality

Mortality	False							True						
**Hospital**	**1(A)**	**2(A)**	**3(A)**	**4(C)**	**5(C)**	**6(A)**	**7(A)**	**1(A)**	**2(A)**	**3(A)**	**4(C)**	**5(C)**	**6(A)**	**7(A)**
**A00_B99**	0.086	0.065	0.065	0.078	0.082	0.083	0.072	0.152	0.115	0.099	0.135	0.112	0.119	0.129
**C00_D49**	0.065	0.059	0.039	0.036	0.029	0.066	0.035	0.177	0.125	0.111	0.080	0.073	0.190	0.102
**D50_D89**	0.032	0.018	0.019	0.015	0.016	0.048	0.018	0.011	0.004	0.003	0.004	0.003	0.015	0.007
**E00_E89**	0.063	0.050	0.063	0.058	0.051	0.055	0.066	0.017	0.025	0.019	0.028	0.018	0.020	0.023
**F01_F99**	0.047	0.047	0.053	0.052	0.072	0.026	0.057	0.020	0.032	0.031	0.030	0.048	0.013	0.023
**G00_G99**	0.038	0.051	0.038	0.065	0.039	0.026	0.028	0.011	0.031	0.028	0.028	0.031	0.008	0.012
**H00_H59**	0.004	0.006	0.003	0.005	0.001	0.001	0.002	NA	NA	NA	NA	NA	NA	NA
**H60_H95**	0.006	0.006	0.004	0.012	0.009	0.006	0.006	NA	NA	NA	NA	NA	NA	NA
**I00_I99**	0.101	0.147	0.143	0.138	0.219	0.138	0.131	0.103	0.211	0.165	0.175	0.249	0.119	0.158
**J00_J99**	0.127	0.132	0.144	0.130	0.106	0.137	0.163	0.217	0.238	0.175	0.256	0.229	0.197	0.258
**K00_K95**	0.108	0.103	0.118	0.059	0.046	0.106	0.094	0.051	0.081	0.079	0.048	0.039	0.085	0.064
**L00_L99**	0.028	0.026	0.029	0.024	0.021	0.022	0.022	0.003	0.005	0.003	0.004	0.006	0.004	0.001
**M00_M99**	0.044	0.041	0.049	0.053	0.036	0.036	0.043	0.009	0.008	0.011	0.011	0.007	0.006	0.006
**N00_N99**	0.059	0.066	0.062	0.076	0.085	0.057	0.063	0.025	0.042	0.030	0.054	0.057	0.035	0.039
**O00_O99**	0.002	0.001	0.001	0.003	0.001	0.000	0.001	NA	NA	NA	NA	NA	NA	NA
**Q00_Q99**	NA	NA	NA	NA	NA	NA	NA	NA	NA	NA	NA	NA	NA	NA
**R00_R99**	0.094	0.134	0.120	0.113	0.116	0.142	0.129	0.019	0.030	0.028	0.011	0.030	0.036	0.035
**S00_T88**	0.074	0.038	0.041	0.072	0.061	0.036	0.059	0.027	0.023	0.016	0.033	0.031	0.016	0.018
**V00_Y99**	0.002	0.002	0.004	0.005	0.003	0.003	0.004	0.004	0.003	0.004	0.013	0.011	0.005	0.009
**Z00_Z99**	0.020	0.008	0.004	0.006	0.005	0.011	0.008	0.156	0.027	0.197	0.089	0.054	0.131	0.117

Values for groupings of diagnosis codes with <= 5 patient encounters have been omitted due to privacy preserving practices required by GEMINI. A = academic hospital, C = community hospital.

© 2025 Subasri V et al. *JAMA Network Open*.

eTable 9. Patient Admission and Stay Characteristics by Hospital and Mortality Status

Mortality	False							True						
Hospital	1(A)	2(A)	3(A)	4(C)	5(C)	6(A)	7(A)	1(A)	2(A)	3(A)	4(C)	5(C)	6(A)	7(A)
# of Encounters	16620	27394	16100	15096	22691	16238	14072	2405	2306	1589	1415	3524	1791	1808
LOS (days)	8.47	8.44	7.90	8.73	10.05	7.55	9.03	14.59	17.19	17.62	19.02	17.72	12.80	14.12
# of Previous Encounters from 2010-2020	0.72	0.60	1.19	0.68	0.60	0.82	0.90	1.28	1.12	1.53	1.36	1.16	1.31	1.63
From Acute Care (%)	2	0	1	1	0	1	1	7	1	1	3	0	1	0
From Nursing Home (%)	6	8	4	11	12	4	10	13	18	14	31	30	8	22
Palliative Care (n)	260	151	50	52	39	160	80	373	56	313	123	191	235	210

Number of patient encounters, average length of stay (LOS), average number of previous encounters from 2010-2020, percentage of patient encounters from acute care institutions, percentage of patient encounters from nursing homes, and number of patients receiving palliative care for their most responsible diagnosis, across hospitals and mortality status. A = academic hospital, C = community hospital.

© 2025 Subasri V et al. *JAMA Network Open*.

eMethods

Clinical Data Shift Experiments

Source data for each experiment was determined based on the nature of the shift (i.e. general, location-based or time-based). In clinical settings, it is common for models to be developed at a given hospital site or time point, and evaluated at a different site or later time point; this would imply that the target data has not been seen, and cannot overlap with the source. However, for general patient characteristics, this is not the case - the source and target data can overlap in age, sex, and admission source, at a given site or timepoint.

General Patient Characteristics

Source (ID): The source dataset for experiments of general patient characteristics is data from the original context/setting of the algorithm, which includes all patient types:
Admitted from nursing home - *Target (OOD)*: Patients admitted from nursing homes.
Admitted from acute care institution - *Target (OOD)*: Patients admitted from acute care institutions.
Sex - *Target (OOD)*: Patients that are i) males ii) females.
Age - *Target (OOD)*: Patients that are i) 18-29 years ii) 30-44 years iii) 45-64 years iv) 65+ years.

Location-Based Shifts

Hospital Type Community - *Source (ID):* Academic hospitals (Hospital 1, 2, 3, 6, 7). *Target (OOD):* Community hospitals (Hospital 4, 5).
Hospital Type Academic - *Source (ID):* Community hospitals (Hospital 4, 5). *Target (OOD)*: Academic hospitals (Hospital 1, 2, 3, 6, 7).

Time-Based Shifts

COVID-19 Pandemic - *Source (ID):* Patients admitted prior to 2020-02-01. *Target (OOD):* Patients admitted from 2020-03-01 to 2020-08-01.
Troponin - *Source (ID):* Patients admitted to Hospitals 6 and 7 prior to 2015-02-01. *Target (OOD):* Patients admitted to Hospitals 6 and 7 from 2015-03-01 to 2015-09-01.
BNP - *Source (ID):* Patients admitted to Hospital 2 prior to 2018-01-01. *Target (OOD):* Patients admitted to Hospital 2 from 2018-02-01 to 2019-01-01.
D-dimer - *Source (ID):* Patients admitted to Hospital 2 prior to 2015-05-01. *Target (OOD):* Patients admitted to Hospital 2 from 2015-06-01 to 2015-12-31.

Sample sizes of training and test cohorts used for shift experiments can be found in **eTable 7**. All reported performance results are from the test set.

© 2025 Subasri V et al. *JAMA Network Open*.
