# Supplementary material for: Detecting and Remediating Harmful Data Shifts for the Responsible Deployment of Clinical AI Models
Source: JAMA Netw Open. 2025 Jun 4;8(6):e2513685. doi: 10.1001/jamanetworkopen.2025.13685 (PMC12138723; doi:10.1001/jamanetworkopen.2025.13685)
Supplement: Supplement 2. — Data Sharing Statement [file jamanetwopen-e2513685-s002.pdf]

Data Sharing Statement

Subasri. Detecting and Remediating Harmful Data Shifts for the Responsible Deployment of Clinical AI Models. *JAMA Netw Open*. Published June 04, 2025.
doi:10.1001/jamanetworkopen.2025.13685

Data
Data available: No

Additional Information
Explanation for why data not available: Data access for research purposes can be requested through the GEMINI network by submitting a Project Proposal Form for review. Further information is available at https://geminimedicine.ca/access-gemini-data/. The code used to perform the experiments in this study can be found at https://github.com/vsubasri/GEMINI-data-shift. We have also provided our monitoring pipeline as part of a broader MLOps framework, to help facilitate the research and deployment of ML models in the clinical setting (https://github.com/VectorInstitute/cyclops).
